# A surface-modified antiperovskite as an electrocatalyst for water oxidation

**DOI:** 10.1038/s41467-018-04682-y

**Published:** 2018-06-13

**Authors:** Yanping Zhu, Gao Chen, Yijun Zhong, Yubo Chen, Nana Ma, Wei Zhou, Zongping Shao

**Affiliations:** 1 State Key Laboratory of Materials-Oriented Chemical Engineering, College of Chemical Engineering, Nanjing Tech University, Nanjing, 210009 China; 2 Department of Chemical Engineering, Curtin University, Perth, 6845 West Australia Australia; 3 School of Materials Science and Engineering, Nanyang Technological University, 50 Nanyang Avenue, Singapore, 639798 Singapore

## Abstract

An efficient and cost-effective oxygen evolution reaction (OER) electrocatalyst is key for electrochemical energy generation and storage technologies. Here, the rational design and in situ formation of an antiperovskite-based hybrid with a porous conductive Cu_1−*x*_NNi_3−*y*_ (*x* and *y* represent defect) core and amorphous FeNiCu (oxy)hydroxide shell is reported as a promising water oxidation electrocatalyst, showing outstanding performance. Benefiting from the unique advantage of core–shell structure, as well as the synergistic effect of Fe, Ni, and Cu and the highly porous hierarchical structure, the hybrid catalyst exhibits highly efficient and robust OER performance in alkaline environments, outperforming the benchmark IrO_2_ catalyst in several aspects. Our findings demonstrate the application potential of antiperovskite-based materials in the field of electrocatalysis, which may inspire insights into the development of novel materials for energy generation and storage applications.

## Abstract (web summary)

Splitting water into its component elements, oxygen and hydrogen gas, provides a carbon-neutral fuel source, although the availability of cheap, earth-abundant catalysts is lacking. Here, the authors demonstrate antiperovskite-derived materials as high-performance water oxidation electrocatalysts.

## Introduction

Oxygen-evolution reaction (OER) electrocatalysts play significant roles in various energy generation and storage processes. The reaction involves a multistep electron transfer process and has intrinsically sluggish kinetics, thus requiring a catalyst for promotion^1–4^. To date, although precious metal oxides (RuO_2_ and IrO_2_) have demonstrated favorable OER activity, the high costs and insufficient stability greatly prohibit their practical applications^1^. Therefore, it is highly desirable to design efficient OER catalysts based on more cost-effective elements.

As alternative OER catalysts, low-cost perovskite-type oxides (ABO_3_; A=rare- and/or alkaline earth metals, B=transition metals) have recently drawn particular attention. These oxides can possess rich combinations of transition metals in their structures, and the A and/or B cations can be substituted by ions of different radii and valences, resulting in flexible structures and tunable OER behaviors^5–8^. However, perovskite oxide-based electrocatalysts have three main drawbacks: large initial overpotential for driving OERs, poor mass activity resulting from the low specific surface area, and poor conductivity at room temperature. Compared with perovskite oxides, transition-metal (sub)nitrides (such as Ni_3_N^9^, Co_4_N^10^, and Ta_5_N_6_^11^) have been reported to show high electrical conductivity and more favorable activities for oxygen evolution. Owing to these facts, novel OER catalysts featuring the great structural flexibility of perovskite oxides, as well as the superior conductivity of transition-metal (sub)nitrides are highly expected.

Interestingly, transitional metal elements and nitrogen/carbon can form an antiperovskite structure at a proper element ratio (AXM_3_; A=Cu, Al, Zn, etc.; X=N or C; M=Ni, Fe, Co, etc.), in which the position of the cations and anions is converted with respect to the conventional perovskite oxides. Due to the high flexibility in elemental composition of perovskite, antiperovskites can also be well tailored to demonstrate versatile properties. Actually, antiperovskites have gained wide attention because of their simple structure and diverse physical properties, including their superconductivity, giant magneto resistance, temperature-invariant resistance and Li-cathode materials^12–15^. Among the various antiperovskite compounds, antiperovskite nitrides ANM_3_ are a very important branch. Experimentally, Ni-based nitride ANNi_3_ (A=Cu and Zn) shows remarkable superconductivity^16^. Theoretically, density functional theory (DFT) calculations reveal that the density of states (DOS) of ANNi_3_ (A=Zn and Cu) is continuous near the Fermi level, suggesting their intrinsically metallic characterization^17^. This is significantly different from perovskite oxides, which are typically insulators at room temperature. Although combining the advantages of perovskite structure and superior conductivity, antiperovskite ANNi_3_ has rarely been employed in the field of electrocatalysis.

Here, we propose an antiperovskite-based porous Cu_1−*x*_NNi_3−*y*_/FeNiCu (oxy)hydroxide core–shell structured hybrid (denoted as p-Cu_1−*x*_NNi_3−*y*_/FeNiCu, with *x* and *y* representing defect) as a high-performance OER electrocatalyst demonstrating excellent activity and durability. A methodology based on the in situ interfacial reaction between CuNNi_3_ and Fe^3+^ for the formation of this core–shell-structured hybrid has been developed, which also leads to the in situ-formed amorphous FeNiCu shell, as well as the formation of the porous architecture. The hybrid exhibits enhanced activity and robust stability compared with the benchmark IrO_2_ electrocatalysts in several aspects.

## Results

### Catalyst synthesis and characterizations

**Fig. 1:**
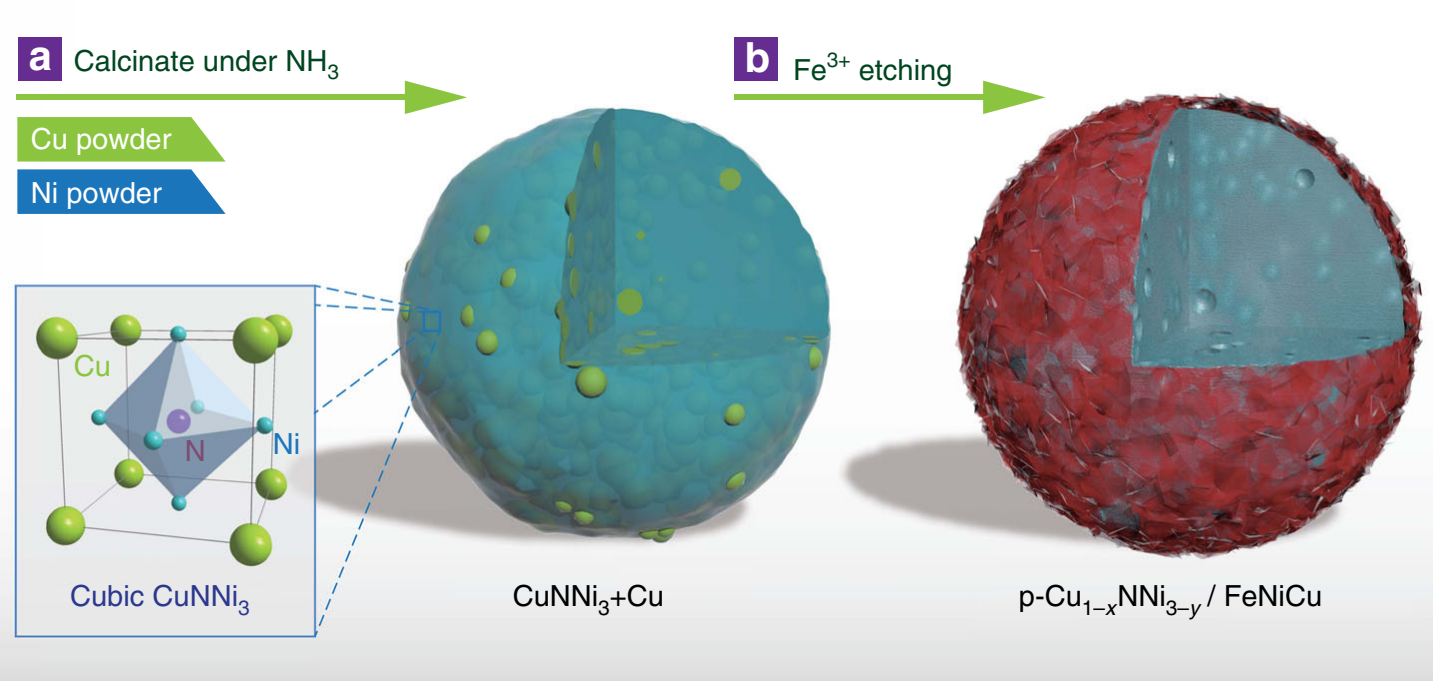
Illustration of the synthesis of p-Cu_1−*x*_NNi_3−*y*_/FeNiCu. **a** Synthesis of CuNNi_3_+Cu through solid-gas reactions. **b** Formation of p-Cu_1−*x*_NNi_3−*y*_/FeNiCu after the Fe^3+^ treatment

The fabrication procedure of the p-Cu_1−*x*_NNi_3−*y*_/FeNiCu hybrid is schematically illustrated in Fig. 1. First, polycrystalline CuNNi_3_ was synthesized from a mixture of copper (Cu) and nickel (Ni) powders through solid–gas reactions. Notably, excessive Cu was intentionally introduced to serve as a sacrificial template for the creation of rich pores in the final hybrid during the following etching process. The as-obtained Cu-excess CuNiN_3_ (denoted as CuNNi_3_+Cu) was then immersed in an FeCl_3_·6H_2_O aqueous solution and kept for 30 min under room temperature. During this process, copper nanoparticles were dissolved according to chemical reaction (1)^18^. Simultaneously, metal-state Cu and Ni in the antiperovskite structure were partially etched by Fe^3+^ (reaction (1) and (2)). Both contributed to the introduction of pores in the antiperovskite phase. Significantly, Fe^3+^, Ni^2+^, and Cu^2+^ ions were hydrolyzed to form an FeNiCu (oxy)hydroxide colloid and H^+^ (reaction (3))^19,20^. Due to the consumption of H^+^ by the dissolution of the antiperovskite, the hydrolysis was promoted. As a result, the precipitated FeNiCu was deposited on the surface of the antiperovskite phase, leading to the formation of a p-Cu_1−*x*_NNi_3−*y*_/FeNiCu core–shell hybrid.

1 $${\mathrm{Cu + 2Fe}}^{3 + } \to {\mathrm{Cu}}^{{\mathrm{2}} + } + {\mathrm{2Fe}}^{{\mathrm{2 + }}}$$

2 $${\mathrm{Ni}} + {\mathrm{2Fe}}^{{\mathrm{3}} + } \to {\mathrm{Ni}}^{{\mathrm{2}} + } + {\mathrm{2Fe}}^{{\mathrm{2}} + }$$

3 $${\mathrm{Fe}}^{{\mathrm{3}} + } + {\mathrm{Ni}}^{{\mathrm{2}} + } + {\mathrm{Cu}}^{{\mathrm{2}} + } + {\mathrm{OH}}^{\mathrm{ - }} \to {\mathrm{FeNiCu}}\,\left( {{\mathrm{oxy}}} \right){\mathrm{hydroxide}} + {\mathrm{H}}^ +$$

**Fig. 2:**
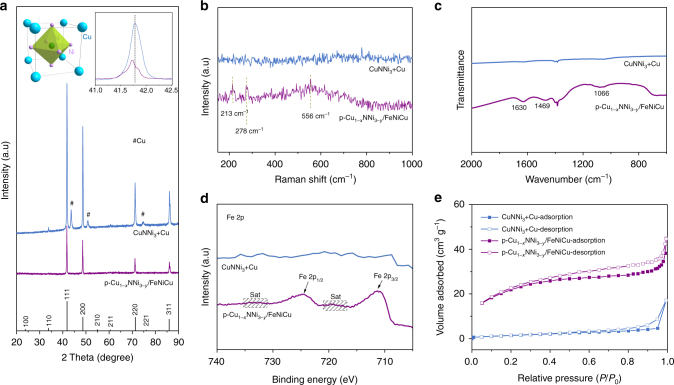
Characterization of catalysts. **a** XRD patterns of the CuNNi_3_+Cu and p-Cu_1−*x*_NNi_3−*y*_/FeNiCu samples. The insets show the unit cell of antiperovskite CuNNi_3_ and the magnified XRD pattern in the 2-theta range of 41–42.5°. **b** Raman spectra, **c** FTIR, **d** XPS spectra of Fe 2p, and **e** N_2_ adsorption–desorption isotherms of the CuNNi_3_+Cu and p-Cu_1−*x*_NNi_3−*y*_/FeNiCu

To reveal the structure and composition of the various samples, X-ray diffraction (XRD) measurement was first carried out. As shown in Fig. 2a, the CuNNi_3_+Cu sample displayed three peaks at 2-theta = 43.1°, 50.2°, and 73.9°, which are corresponded to the metallic Cu phase. The remaining strong diffraction peaks indexed well with a cubic antiperovskite cell^17^. Rietveld refinement confirms the cubic structure (space group: *pm3̅m*) with a lattice constant of *a* = *b* = *c* = 3.741(5) Å (Supplementary Fig. 1a, Supplementary Table 1), which agrees well with that (3.745 Å) predicted by first-principle calculations^16^. The corresponding antiperovskite crystal structure of CuNNi_3_ is shown in the inset of Fig. 2a. After etching by Fe^3+^ for 30 min, the three peaks assigned to the metallic Cu were not observed, suggesting that the metallic Cu redundant was successfully dissolved. The new diffraction pattern still overlapped satisfactorily with that of the cubic antiperovskite phase (Rietveld refinement in Supplementary Fig. 1b; Supplementary Table 1), but with a reduction in peak intensity and slightly negative peak shift compared with the CuNNi_3_+Cu sample. It is assumed that the FeCl_3_ aqueous solution not only etched the excess metallic Cu but also influenced the antiperovskite. No additional peaks assigned to Fe-related phases were observed. To gain more information about the nature of the Fe^3+^-treated antiperovskite, the samples were then characterized using Raman spectroscopy. Compared with the CuNNi_3_+Cu sample, three additional Raman bands (Fig. 2b) located at 213 cm^−1^, 278 cm^−1^, and 556 cm^−1^ appeared, which are derived from asymmetric stretching of the metal and (oxy)hydroxide groups^20–22^. Similarly, the bands at 1630 cm^−1^, 1469 cm^−1^, and 1066 cm^−1^ in the Fourier transform infrared (FTIR) spectrum of the Fe^3+^-treated antiperovskite (Fig. 2c) can be ascribed to the metal-O vibrational modes^20^. X-ray photoelectron spectroscopy (XPS) analyses were further performed to analyze the chemical composition of the samples. The full-survey spectrum (Supplementary Fig. 2a) of the treated antiperovskite shows distinct peaks identifying the Fe elements^23^. The core-level spectrum of Fe 2p (Fig. 2d) displays peaks at 724.6 eV and 711.6 eV for Fe 2p_1/2_ and Fe 2p_3/2_, respectively^24^, together with two satellite lines that indicate the presence of Fe^3+^. Based on the aforementioned evidence, the deposition of amorphous FeNiCu (oxy)hydroxide on the treated antiperovskite was readily confirmed. Moreover, the N 1 s spectrum of the CuNNi_3_+Cu displays a sharp peak at 397.7 eV (Supplementary Fig. 2b), which is ascribed to the metal-N bond^25^. After the Fe^3+^ treatment, the peak of the p-Cu_1−*x*_NNi_3−*y*_/FeNiCu decreased in intensity because of the presence of the FeNiCu shell layer on the surface of Cu_1−*x*_NNi_3−*y*_.

Nitrogen adsorption–desorption measurements were carried out to further investigate the pore structures of the CuNNi_3_+Cu sample and the p-Cu_1−*x*_NNi_3−*y*_/FeNiCu hybrid. As plotted in Fig. 2e, the CuNNi_3_+Cu sample presented a type-III isotherm without hysteresis loops, suggesting a non-porous nature^26^. In contrast, the sample after the Fe^3+^ treatment possessed a type-IV isotherm with a large hysteresis loop, indicating the presence of rich mesopores^27^. Notably, the p-Cu_1−*x*_NNi_3−*y*_/FeNiCu hybrid exhibited a specific surface area (SSA) of 76.7 m^2^ g^−1^ and a total pore volume (V_Total_) of 0.095 cm^3^ g^−1^, which are much larger than those of the CuNNi_3_+Cu sample (5.2 m^2^ g^−1^ and 0.004 cm^3^ g^−1^). Moreover, based on the isotherms, the pore size distribution of p-Cu_1−*x*_NNi_3−*y*_/FeNiCu was over a relatively wide range (Supplementary Fig. 3). To emphasize the importance of the excessive Cu in the CuNNi_3_ + Cu sample, two reference samples were prepared: a pure-phase antiperovskite prepared from stoichiometric Cu and Ni (1:3, denoted as CuNNi_3_), and the Fe^3+^-treated CuNNi_3_ (defined as Cu_1−*x*_NNi_3−*y*_/FeNiCu, Supplementary Fig. 4). The specific surface areas of CuNNi_3_ and Cu_1−*x*_NNi_3−*y*_/FeNiCu were also measured. According to the nitrogen adsorption–desorption isotherms (Supplementary Fig. 5), the Cu_1−*x*_NNi_3−*y*_/FeNiCu had a smaller hysteresis loop with a lower SSA value of 36.9 m^2^ g^−1^, which was only half that of p-Cu_1−*x*_NNi_3−*y*_/FeNiCu. Clearly, the introduction of excessive Cu during synthesis effectively facilitated the formation of a porous structure with rich mesopores. The highly porous structure allows a large electrode/electrolyte contact area^26,27^, which is beneficial to the charge and mass transfer during the electrocatalysis.

**Fig. 3:**
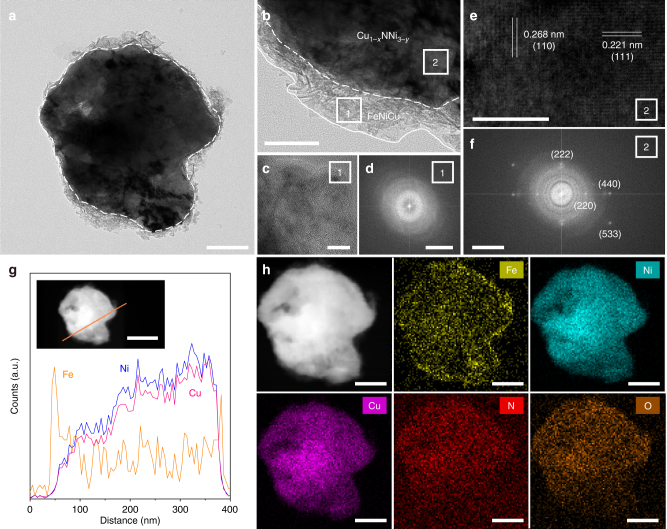
Morphology and texture characterization. **a** TEM and **b** magnified TEM images of p-Cu_1−*x*_NNi_3−*y*_/FeNiCu. **c** HRTEM and **d** the corresponding FFT images of the FeNiCu shell. **e** HRTEM and **f** the corresponding FFT images of the Cu_1−*x*_NNi_3−*y*_ core. **g** Line-scan EDS spectra and **h** STEM-EDS element mapping images of the p-Cu_1−*x*_NNi_3−*y*_/FeNiCu. Scale bar in **a** is 100 nm, in **b** is 50 nm, in **c** is 10 nm, in **d** is 5 nm^−1^, in **e** is 5 nm, in **f** is 5 nm^−1^, in **g** is 200 nm and in **h** is 100 nm

The general morphology and textural details of the catalysts were reflected by field-emission scanning electron microscopy (FESEM) and transmission electron microscopy (TEM). Similar to traditional perovskites, the CuNNi_3_+Cu sample shows serious agglomeration of particles several hundred nanometers in size (Supplementary Fig. 6)^6,8^. The representative TEM image with close observation (Supplementary Fig. 7a and b) shows a continuously smooth surface of the antiperovskite and the HRTEM image in Supplementary Fig. 7c gives lattice spacings of 0.264 nm and 0.210 nm corresponding to the (110) and (111) interplanar spacings of CuNNi_3_ and Cu, respectively. In addition, the energy disperse spectrum (EDS) elemental mapping (Supplementary Fig. 7d) further confirms that the elements of Cu, Ni, and N were homogeneously distributed in the selected field. Applying Fe^3+^ treatment to the Cu-excess CuNNi_3_ sample caused a large change in the morphology. The p-Cu_1−*x*_NNi_3−*y*_/FeNiCu showed a loose nature (Supplementary Fig. 8). TEM investigations (Fig. 3a) further revealed that thin nanoflakes were decorated on the initially smooth surface of the antiperovskite, demonstrating a typical core–shell structure. The EDS spectra obtained from core and shell confirmed that the elements of the shell layer consist of Fe, Ni, and Cu (Supplementary Fig. 9). Close observation, shown in Fig. 3b, displays a distinguished Cu_1−*x*_NNi_3−*y*_–FeNiCu interface (as indicated by a white dashed line). The HRTEM image (Fig. 3c) of the FeNiCu shell and the associated fast Fourier transform (FFT) pattern of the image (Fig. 3d) are characteristic of an amorphous phase. Conversely, the lattice spacing of the Cu_1−*x*_NNi_3−*y*_ core in the HRTEM image (Fig. 3e) was determined to be 0.268 nm and 0.221 nm, which corresponds to the (110) and (111) planes of cubic CuNNi_3_, respectively, and the FFT pattern (Fig. 3f) with bright spots depicts the polycrystalline nature of CuNNi_3_. EDS line-scan spectra and elemental mapping analysis further identify the quintessential core–shell structure. As shown in Fig. 3g, Fe possesses strong signals in the shell region, and from Fig. 3h, Fe was located across the entire area with strong signals at the edges.

**Fig. 4:**
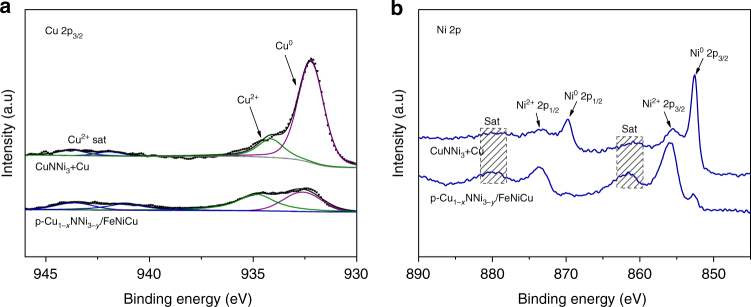
XPS characterization. **a** Cu 2p_3/2_ and **b** Ni 2p XPS spectra of the CuNNi_3_+Cu and p-Cu_1−*x*_NNi_3−*y*_/FeNiCu

To analyze the surface chemical valences of the samples before and after Fe^3+^ treatment, Fig. 4 compares the XPS spectra of the CuNNi_3_+Cu sample and the p-Cu_1−*x*_NNi_3−*y*_/FeNiCu hybrid in the Cu 2p_3/2_ and Ni 2p regions. For the CuNNi_3_+Cu sample, two contributions of Cu are observed at 934.1 and 932.2 eV (Fig. 4a). The higher binding energy (BE) peak can be indexed to Cu^2+^, accompanied by the characteristic shakeup satellite peaks (941.9 eV and 943.7 eV). The lower BE peak indicates the presence of Cu^0^ or Cu^+^ species^25,28^. Because Cu^0^ and Cu^+^ cannot be differentiated in Cu 2p_3/2_ spectra, Auger Cu LMM spectra (Supplementary Fig. 10) were used to verify the presence of metallic Cu^0^ at a BE of ~568.3 eV^28^. After Fe^3+^ treatment, the intensity of the Cu^2+^ and satellite peaks in the p-Cu_1−*x*_NNi_3−*y*_/FeNiCu hybrid is significantly enhanced. Figure 4b shows the core-level lines of Ni. Two distinct peaks at 869.7 eV (2p_1/2_) and 852.5 eV (2p_3/2_) of the CuNNi_3_+Cu emerged as the metallic state of Ni. The other two peaks centered at 873.7 eV and 855.4 eV are characteristic of Ni^2+^ 2p_1/2_ and Ni^2+^ 2p_3/2_, respectively, accompanied by two satellite peaks^29,30^. Similar to the intensity of Cu, after Fe^3+^ treatment, the intensity of divalent Ni significantly increased in p-Cu_1−*x*_NNi_3−*y*_/FeNiCu. Along with the results of Fig. 2d, the surface of the p-Cu_1−*x*_NNi_3−*y*_/FeNiCu hybrid consists primarily of Fe^3+^, Ni^2+^, and Cu^2+^.

### Electrochemical performance

**Fig. 5:**
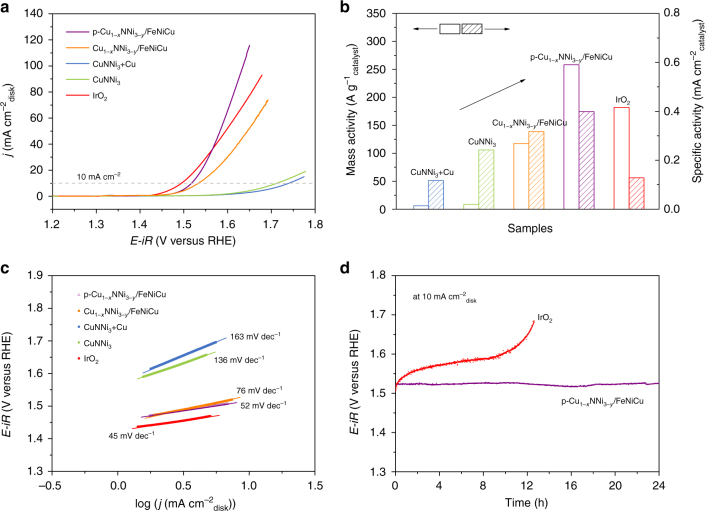
Electrochemical properties of catalysts. **a** Ohmic resistance-corrected OER activity curves of the IrO_2_, CuNNi_3_+Cu, p-Cu_1−*x*_NNi_3−*y*_/FeNiCu, CuNNi_3_ and Cu_1−*x*_NNi_3−*y*_/FeNiCu catalysts in 1 M KOH at 5 mV s^−1^. **b** Mass activities and BET surface area-normalized intrinsic activities of catalysts at *η* = 400 mV derived from **a**. **c** Tafel plots obtained from the steady-state measurements. **d** Chronopotentiometric curves of p-Cu_1−*x*_NNi_3−*y*_/FeNiCu and commercial IrO_2_ at 10 mA cm^−2^_disk_

The performance of the as-obtained p-Cu_1−*x*_NNi_3−*y*_/FeNiCu hybrid as an electrocatalyst toward OER was assessed in 1 M KOH solution adopting a three-electrode configuration. The catalyst mass loading was controlled 0.367 mg cm^−2^. Similar measurements were performed on CuNNi_3_+Cu, CuNNi_3_, Cu_1−*x*_NNi_3−*y*_/FeNiCu and commercial IrO_2_ for direct comparison. All potentials reported were referenced to reversible hydrogen electrode (RHE). The typical polarization curves (after ohmic resistance corrections) of the five electrodes are displayed in Fig. 5a. The IrO_2_ catalyst demonstrated highly active OER performance with the lowest onset potential of 1.43 V and an overpotential of only 260 mV to reach 10 mA cm^−2^ (*η*_10_), which is consistent with the earlier reports^31^. The CuNNi_3_ catalyst exhibited lower onset potential (1.58 V) and a smaller *η*_10_ value (480 mV) compared to those of the Cu-excess CuNNi_3_ sample (CuNNi_3_+Cu, onset potential of 1.60 V, *η*_10_ = 510 mV), indicating that excessive Cu in the CuNNi_3_+Cu sample was less active than the antiperovskite towards the OER. However, after Fe^3+^ treatment, the Cu_1−*x*_NNi_3−*y*_/FeNiCu electrode had a dramatically decreased onset potential of 1.46 V and a reduced overpotential of 300 mV at 10 mA cm^−2^. Furthermore, when p-Cu_1−*x*_NNi_3−*y*_/FeNiCu with an enlarged surface area was used as the OER electrode, the catalytic activity was further enhanced. It needed an overpotential of only 280 mV to drive 10 mA cm^−2^, and the catalytic current largely exceeded that of the benchmark IrO_2_ in the high overpotential region (above 330 mV). Compared with other low-cost OER catalysts, the p-Cu_1−*x*_NNi_3−*y*_/FeNiCu hybrid exhibits competitive catalytic performance (Supplementary Table 4). Figure 5b plots the mass activity (normalized to the mass loading) and specific activity (normalized to the surface area from BET measurements^1,32^ (Supplementary Fig. 11, Supplementary Table 2)) of the various electrocatalysts at *η* = 400 mV (1.63 V vs RHE). The p-Cu_1−*x*_NNi_3−*y*_/FeNiCu electrocatalyst exhibited a mass activity of 258.3 Ag^−1^, which is ~2.2 and 1.5 times that of the Cu_1−*x*_NNi_3−*y*_/FeNiCu and IrO_2_ electrocatalysts, respectively. The specific activity is well known to reflect the intrinsic activity of a catalyst for the OER^6,7^. From Fig. 5b, the intrinsic OER activity of catalysts followed the order of CuNNi_3_+Cu<IrO_2_<CuNNi_3_<Cu_1−*x*_NNi_3−*y*_/FeNiCu<p-Cu_1−*x*_NNi_3−*y*_/FeNiCu, indicating the attractive intrinsic activity of antiperovskite and again suggesting the highest OER activity for the p-Cu_1−*x*_NNi_3−*y*_/FeNiCu electrocatalyst.

Tafel plots were derived from polarization curves to gain the kinetic parameters of the electrodes. As drawn in Fig. 5c, the p-Cu_1−*x*_NNi_3−*y*_/FeNiCu electrocatalyst had a Tafel slope of ~52 mV dec^−1^, which is close to that of IrO_2_ (45 mV dec^−1^) but much smaller than other catalysts (76–163 mV dec^−1^), indicating that the rate-determining step of the p-Cu_1−*x*_NNi_3−*y*_/FeNiCu electrocatalyst occurs near the end of the multiple-electron transfer reaction, which is commonly a sign of an efficient electrocatalyst^33^. Further, we compared the electrochemical surface area (ECSA) of each catalyst, which was obtained from the double-layer capacitance (C_dl_) (Supplementary Fig. 12). It was found that the p-Cu_1−*x*_NNi_3−*y*_/FeNiCu catalyst exhibited the largest C_dl_ (10.5 mF cm^−2^) among all four samples, demonstrating the highest electrochemically active surface area^29–31^. The polarization curves of the catalysts normalized by ECSA are displayed in Supplementary Fig. 13. Obviously, the p-Cu_1−*x*_NNi_3−*y*_/FeNiCu still showed the best OER activity among the catalysts. In addition, electrochemical impedance spectroscopy (EIS) measurements were performed to demonstrate the significant electrode kinetic of the p-Cu_1−*x*_NNi_3−*y*_/FeNiCu. Nyquist plots were depicted in Supplementary Fig. 14 for the CuNNi_3_+Cu, CuNNi_3_, Cu_1−*x*_NNi_3−*y*_/FeNiCu and p-Cu_1−*x*_NNi_3−*y*_/FeNiCu electrodes. The p-Cu_1−*x*_NNi_3−*y*_/FeNiCu catalyst had a much lower charge–transfer resistance (*R*_ct_) value, corresponding to a rate of reaction that was faster than those of the other samples^8^.

Long-term stability is another important criterion for a good OER catalyst. Figure 5d shows the chronopotentiometric curves for p-Cu_1−*x*_NNi_3−*y*_/FeNiCu and IrO_2_ at a fixed current density of 10 mA cm^−2^. Apparently, the potential from the IrO_2_ catalyst increased gradually resulting from the formation of a soluble complex anion IrO_4_^2−^ in alkaline environments^33^. In contrast, the potential from p-Cu_1−*x*_NNi_3−*y*_/FeNiCu almost remained unchanged throughout the entire 24-h test, indicating a high stability of the p-Cu_1−*x*_NNi_3−*y*_/FeNiCu catalyst.

## Discussion

The unique advantage of core–shell structure of the p-Cu_1−*x*_NNi_3−*y*_/FeNiCu hybrid plays a vital role in enhancing the catalytic performance. The metallic antiperovskite core contributes high electrical conductivity, which enhances the facile electron transfer during the catalytic process. The metal (oxy)hydroxide shell is formed on surface of the metallic core, which makes the shell the active catalyst. The two components contribute synergistically during the OER process. Besides, the interaction of Fe, Ni, and Cu in the p-Cu_1−*x*_NNi_3−*y*_/FeNiCu needs to be verified. It is well known that the doping Fe in Ni-based catalysts can enhance the catalytic activity^34–36^. According to the aforementioned Tafel slope analysis, the Tafel slope is crucially affected by Fe^3+^. This result strongly supports the conclusion that doping Fe into NiCu based materials can adjust the catalytic property by tuning the intrinsic kinetics^37,38^. To determine the role of Cu, cyclic voltammograms of p-Cu_1−*x*_NNi_3−*y*_/FeNiCu and Cu_1−*x*_NNi_3−*y*_/FeNiCu electrodes were recorded (Supplementary Fig. 15). ICP-OES results (Supplementary Table 3) revealed that the two samples have similar Fe content, while the content of Cu in the shell layer of p-Cu_1−*x*_NNi_3−*y*_/FeNiCu is higher than that of Cu_1−*x*_NNi_3−*y*_/FeNiCu. Obviously, the Ni^2+^/Ni^3+^ oxidation peak of the p-Cu_1−*x*_NNi_3−*y*_/FeNiCu shifted to a negative potential relative to that for the Cu_1−*x*_NNi_3−*y*_/FeNiCu, suggesting that the electrochemical oxidation of Ni^2+^ to Ni^3+^ is facilitated by the larger concentration of Cu^39^. From the above results, the high catalytic activity p-Cu_1−*x*_NNi_3−*y*_/FeNiCu is also derived from the synergistic effect of Fe, Ni and Cu. Finally, the highly porous structure provided large amounts of available active sites, thus further enhancing the catalytic performance.

Ex situ XPS measurements were conducted to investigate the compositional change of p-Cu_1−x_NNi_3−y_/FeNiCu before and after the OER electrolysis. The divalent Cu peak in the Cu 2p_3/2_ spectrum (Supplementary Fig. 16a) slightly increased in intensity after the OER. For the Ni 2p spectrum (Supplementary Fig. 16b), the formation of Ni^III^ is in accordance with previous reports^40–42^. The post-OER Fe 2p XPS spectrum (Supplementary Fig. 16c) has the same shape as that of the p-Cu_1−*x*_NNi_3−*y*_/FeNiCu catalyst.

To optimize the OER performance, the ratio of the antiperovskite Cu_1−*x*_NNi_3−*y*_ core to the amorphous FeNiCu shell was optimized in control experiments by varying the time of the Fe^3+^ treatment. Four other catalysts under different immersion times were prepared and are designated Catalyst_5 min_, Catalyst_15 min_, Catalyst_60 min_, and Catalyst_120 min_. Supplementary Fig. 17a displays the XRD patterns of the samples with different times. As the treatment time was prolonged, the diffraction peaks assigned to the antiperovskite phase significantly decreased in intensity, which was caused by the gradual dissolution of the antiperovskite in the H^+^ environment. Meanwhile, as more H^+^ was consumed by the antiperovskite, the hydrolysis process was further promoted. As expected, the Catalyst_120 min_ had the most obvious Raman peaks indexed to precipitated FeNiCu deposited on the antiperovskite (Supplementary Fig. 17b). As revealed by the corresponding SEM images (Supplementary Fig. 18), the samples became increasingly flocculent with elongated treatment time. The OER activities of all four samples with different treatment times were evaluated under the same condition (Supplementary Fig. 19a). The Catalyst_30 min_ electrode displayed the lowest onset potential and the smallest overpotential to produce 10 mA cm^−2^. After normalizing to the mass loading and the surface area from BET measurements, the Catalyst_30 min_ still showed the best mass activity and intrinsic activity among all the catalysts (Supplementary Figs. 19b and 20, Supplementary Table 2).

In control experiments, the role of the antiperovskite Cu_1−*x*_NNi_3−*y*_ in the p-Cu_1−*x*_NNi_3−*y*_/FeNiCu core–shell hybrid was studied. The CuNi alloy (denoted as CuNi) was first prepared via the similar procedure with CuNNi_3_+Cu except replacing the ammonia atmosphere with 10% H_2_/Ar. The conductivity of CuNi was determined to be about 2.5 × 10^3^ S cm^−1^, examined by a four-probe electrical measurement, which is larger than that of the CuNNi_3_+Cu (1.62 × 10^3^ S cm^−1^). XRD patterns of CuNi before and after the immersion in Fe^3+^ (Supplementary Fig. 21a) revealed that the conductive CuNi substrate is stable during the treatment. The EDS spectra obtained from core and shell confirmed that the shell layer consists of Fe, Ni and Cu (Supplementary Fig. 21b & c), and the amorphous shell and the crystalline core of the Fe^3+^-CuNi are similar to those of the p-Cu_1−*x*_NNi_3−*y*_/FeNiCu (Supplementary Fig. 21d-g). XPS spectra of the CuNi and Fe^3+^-CuNi in the Cu 2p_3/2_, Ni 2p and Fe 2p regions (Supplementary Fig. 22a-c) confirmed that the surface of the Fe^3+^-CuNi hybrid consists primarily of Fe^3+^, Ni^2+^, and Cu^2+^. Moreover, the SSA values of the CuNi (4.6 m^2^ g^−1^) and Fe^3+^-CuNi (69.9 m^2^ g^−1^) are close to those of the CuNNi_3_+Cu (5.2 m^2^ g^−1^) and the p-Cu_1−*x*_NNi_3−*y*_/FeNiCu (76.7 m^2^ g^−1^), respectively (Supplementary Fig. 22d). Although the Fe^3+^-CuNi sample has a structure similar to that of p-Cu_1−*x*_NNi_3−*y*_/FeNiCu except the core, when serving as an OER catalyst (Supplementary Fig. 23), the Fe^3+^-CuNi electrode still shows far inferior performance compared to the p-Cu_1−*x*_NNi_3−*y*_/FeNiCu, confirming that the superior catalytic performance of the p-Cu_1−*x*_NNi_3−*y*_/FeNiCu hybrid is closely dependent on the antiperovskite core, not merely derived from the FeNiCu layer. The atom arrangement in the alloy core of the Fe^3+^-CuNi is disordered while the Cu_1−*x*_NNi_3−*y*_ core has an definite atom arrangement due to the antiperovskite structure, which can facilitate the adsorption of OH^−^, thus promoting the OER activity.^43^ Besides, the Ni and Cu in the antiperovskite exist in form of Ni^0^, Ni^2+^ and Cu^0^, Cu^2+^, respectively, while the Ni^2+^ and Cu^2+^ in the alloy only exist on surface resulting from the partially surface oxidation. It is acknowledged that higher oxidation state Cu^2+^/Ni^2+^ species are more favorable for OER than the Cu^0^/Ni^0^ species.^44^ In this regard, compared to the alloy core, the antiperovskite core possesses unique structural and electronic features which can significantly dominate the OER activity.

We then treated bulk NiO and metallic Ni_3_N with Fe^3+^ under the same condition. Supplementary Fig. 24 compares the XRD patterns of NiO and Ni_3_N before and after the immersion in Fe^3+^, respectively. The peaks assigned to Ni_3_N in the Fe^3+^-Ni_3_N hybrid nearly vanished (Supplementary Fig. 24a), and the intensity of the diffraction peaks of NiO in Fe^3+^-NiO also decreased compared with that of the bulk NiO (Supplementary Fig. 24b). When serving as an OER catalyst (Supplementary Fig. 25), both Fe^3+^-NiO and Fe^3+^-Ni_3_N exhibited enhanced performance compared to those of bulk NiO and metallic Ni_3_N, respectively, confirming that the Fe^3+^ treatment can help to improve the catalytic performance. However, the Fe^3+^-NiO electrode shows far inferior performance compared to p-Cu_1−*x*_NNi_3−*y*_/FeNiCu because the remarkable conductivity of Cu_1−*x*_NNi_3−*y*_ allows significantly more efficient electron transportation compared with the insulating character of NiO^9^. For the Fe^3+^-Ni_3_N and the p-Cu_1−*x*_NNi_3−*y*_/FeNiCu hybrid, both the Ni_3_N and Cu_1−*x*_NNi_3−*y*_ cores are intrinsically metallic^9^. However, Ni_3_N was almost dissolved during Fe^3+^ treatment, while the Cu_1−*x*_NNi_3−*y*_ exhibited the structural stability of the antiperovskite. Furthermore, the representative perovskite oxides Ba_0.5_Sr_0.5_Co_0.8_Fe_0.2_O_3−δ_ (BSCF) and SrTi_0.1_Fe_0.85_Ni_0.05_O_3-δ_ (STFN) were treated by the Fe^3+^. Obviously, the catalytic performance of the p-Cu_1−*x*_NNi_3−*y*_/FeNiCu hybrid outperforms the Fe^3+^-BSCF and Fe^3+^-STFN catalysts (Supplementary Fig. 26). All the above results demonstrated that the antiperovskite core of the p-Cu_1−*x*_NNi_3−*y*_/FeNiCu hybrid not only functions as a conductive and stable substrate, but also plays a vital role in enhancing the catalytic performance.

In summary, an antiperovskite-based hybrid composed of a p-Cu_1−*x*_NNi_3−*y*_ core with a surface modified with a FeNiCu shell has been developed as an efficient OER electrocatalyst. The hybrid achieved excellent OER activity due to the unique advantage of core–shell structure, as well as the synergistic effect of Fe, Ni and Cu and the highly porous hierarchical structure. The antiperovskite core of the p-Cu_1−*x*_NNi_3−*y*_/FeNiCu hybrid not only acts as a conductive substrate, but also plays a vital role in promoting the OER performance during the electrocatalysis. The rational design demonstrated here should be generally applicable to the fabrication of other low-cost OER electrocatalysts. More importantly, the investigation of antiperovskite in an electrochemical application may open a new field and inspire insights into the development of novel materials for energy generation and storage applications.

## Methods

### Synthesis of CuNNi_3_+Cu and p-Cu_1−*x*_NNi_3−*y*_/FeNiCu core–shell hybrid

Typically, 7.5 mM Copper (Cu, 99.9%) and 15 mM Nickel (Ni, 99.9%) powders were thoroughly mixed and ground, followed by heating in a NH_3_ flow (99.9%, 100 mL min^−1^) at 673 K for 3 h in a tube furnace. The as-obtained powders were then reground, pelletized, and repeatedly sintered at 773 K for 6 h and at 833 K for 6 h in the same NH_3_ flow. After being cooled to room temperature (RT) naturally, CuNNi_3_+Cu was finally obtained. Afterwards, 0.3 g of the CuNNi_3_+Cu sample was immersed into a 100 mL aqueous solution containing 1.35 g of FeCl_3_·6H_2_O and stirred under RT for 30 min to synthesize the p-Cu_1−*x*_NNi_3−*y*_/FeNiCu core–shell hybrid.

### Synthesis of CuNNi_3_ and Cu_1−*x*_NNi_3−*y*_/FeNiCu samples

The CuNNi_3_ sample was synthesized by stoichiometric Cu (5 mM, CuO, 99.9%) and nickel (15 mM) powders following the same procedure as that of the CuNNi_3_+Cu. The Cu_1−*x*_NNi_3−*y*_/FeNiCu was prepared from CuNNi_3_ under the same condition as that of the p-Cu_1−*x*_NNi_3−*y*_/FeNiCu.

### Synthesis of Catalyst_5 min_, Catalyst_15 min_, Catalyst_60 min_, and Catalyst_120 min_ samples

In control experiments, samples with different immersion times (denoted as Catalyst_5 min_, Catalyst_15 min_, Catalyst_60 min_ and Catalyst_120 min_) were prepared under the same procedure as that of the p-Cu_1−*x*_NNi_3−*y*_/FeNiCu (Catalyst_30 min_).

### Synthesis of CuNi and Fe^3+^-CuNi

CuNi was produced via the similar method with CuNNi_3_+Cu except replacing the ammonia atmosphere with 10% H_2_/Ar. The Fe^3+^-CuNi was synthesized from CuNi powder following the same method as that of the p-Cu_1−*x*_NNi_3−*y*_/FeNiCu.

### Synthesis of Fe^3+^-NiO

Bulk NiO (99%) was purchased and used as received. The Fe^3+^-NiO was synthesized from NiO powder following the same method as that of the p-Cu_1−*x*_NNi_3−*y*_/FeNiCu.

### Synthesis of bulk Ni_3_N and Fe^3+^-Ni_3_N

Bulk Ni_3_N was produced following a strategy reported previously^9^. Briefly, 1.5 g of Ni(NO_3_)_2_.6H_2_O (5.2 mM) was introduced to 10 mL of ammonia solution (28%) to form a dark blue complex under vigorous stirring. The complex was then heated at 653 K under a flowing NH_3_ (100 mL min^−1^) to obtain the bulk Ni_3_N. The Fe^3+^-Ni_3_N was prepared from Ni_3_N under the same condition as that of the p-Cu_1−*x*_NNi_3−*y*_/FeNiCu.

### Synthesis of bulk Ba_0.5_Sr_0.5_Co_0.8_Fe_0.2_O_3−δ_ (BSCF) and Fe^3+^-BSCF

The perovskite Ba_0.5_Sr_0.5_Co_0.8_Fe_0.2_O_3−δ_ was prepared through the sol–gel method reported previously^6^. First, stoichiometric Ba(NO_3_)_2_, Sr(NO_3_)_2_, Co(NO_3_)_2_·6H_2_O, and Fe(NO_3_)_3_·9H_2_O were added to the deionized water and dissolved under vigorous stirring. Then ethylenediaminetetraacetic acid (C_10_H_16_N_2_O_8_, EDTA) and citric acid (C_6_H_8_O_7_, CA) were introduced as complexing agents to the above solution and the molar ratio of EDTA: CA: metal ions was 1:2:1. In order to promote the complexation process, an ammonium solution was added to adjust the pH of the solution to about 6. The mixture was under vigorous stirring at 90 °C to allow the water to evaporate and yield a gel. The above transparent gel was transferred to a crucible and pre-treated at 250 °C for 5 h to obtain a solid precursor. The BSCF was finally obtained after subsequently calcinated at 1000 °C for 10 h in air. The Fe^3+^-BSCF was prepared from BSCF under the same condition as that of the p-Cu_1−*x*_NNi_3−*y*_/FeNiCu.

### Synthesis of bulk SrTi_0.1_Fe_0.85_Ni_0.05_O_3−δ_ (STFN) and Fe^3+^-STFN

The perovskite SrTi_0.1_Fe_0.85_Ni_0.05_O_3−δ_ was produced through the similar synthesis route with BSCF except that the final calcination was conducted at 1000 °C and held for 5 h. The Fe^3+^-STFN was prepared from STFN under the same condition as that of the p-Cu_1−*x*_NNi_3−*y*_/FeNiCu.

### Materials characterization

RT powder X-ray diffraction (XRD) was performed on the Rigaku Smartlab equipped with Cu kα radiation. The scanning was set in the 2*θ* range from 10° to 90° with intervals of 0.02°. Rietveld refinement of the XRD patterns was conducted to get more detailed structural information of the samples using the GSAS-EXPGUI package. Raman spectra were recorded using a micro-Raman spectrometer (HR800 UV). The Fourier transform infrared (FTIR) spectra were collected from 4000 to 500 cm^−1^ using a spectrophotometer (Nexus 870). X-ray photoelectron spectroscopy (XPS) measurements were performed on PHI550 and the data were fitted by XPSPEAK41. The specific surface areas were characterized through nitrogen adsorption-desorption measurements (BELSORP II) using the Brunauer–Emmett–Teller (BET) method. The corresponding pore size distributions were estimated according to the Barrett–Joyner–Halenda (BJH) method. Approximately 2.0 g of the samples were pretreated under 200 °C for at least 3 h before N_2_ physisorption measurements at 77 K. Field-emission scanning electron microscope (FESEM) images were acquired on a HITACHI S-4800, transmission electron microscope (TEM) and high-resolution transmission electron microscope (HRTEM) images were collected from JEOL JEM-200CX.

### Electrochemical measurements

Electrochemical performance was assessed on a Bio-Logic SP-300 workstation at RT. All tests were carried out in 1 M potassium hydroxide (KOH) electrolyte in a conventional three-electrode configuration. Reference electrode: Ag/AgCl electrode. Counter electrode: platinum wire. Working electrode: catalyst-modified glassy carbon (GC). The catalyst ink contained 10 mg of the measured catalyst, 10 mg of conductive carbon (Super P Li) and 100 μL of 5 wt% Nafion solution ultrasonically dispersed in 1 mL of the absolute ethanol. In total 10-μL of the homogeneous dispersion was transferred onto the surface of the GC substrate (0.2475 cm^2^) and dried at RT. The catalyst mass loading was ~0.367 mg cm^–2^_disk_.

All the potentials reported were referenced vs reversible hydrogen electrode (RHE) according to the equation with IR compensation: E(Ag/AgCl)+1.002 V = E(RHE). The overpotential (*η*) was obtained based on the equation: E(RHE)−1.23 V = *η* (V). The electrolyte was purged with O_2_ for 30 min before each measurement. Polarization curves were recorded using linear sweep voltammetry (LSV) under a rotation rate of 1600 r.p.m. The potential window ranged from 0 to 0.8 V (vs Ag/AgCl) and the scanning rate was 5 mV s^−1^. Cyclic voltammograms (CVs) tests were carried out in the potential window of 0.07–0.17 V (vs. Ag/AgCl) with different sweeping rates from 20 to 100 mV s^−1^ to calculate the electrochemical double-layer capacitances (EDLCs), C_dl_. Electrochemical impedance spectroscopy (EIS) measurements were carried out in the frequency range of 10^5^ to 0.1 Hz at 0.7 V vs. Ag/AgCl. The a.c. voltage was set at 5 mV. Durability was evaluated by chronopotentiometry test performed under constant current density of 10 mA cm^−2^_disk_ with a rotation rate of 1600 r.p.m.

### Data availability

The data that support the findings of this study are available from the corresponding authors upon request.

## Electronic supplementary material

Supplementary Information
