# Supplementary material for: A surface-modified antiperovskite as an electrocatalyst for water oxidation
Source: Nat Commun. 2018 Jun 13;9:2326. doi: 10.1038/s41467-018-04682-y (PMC5997991; doi:10.1038/s41467-018-04682-y)
Supplement: Supplementary file 1 — Supplementary Information [file 41467_2018_4682_MOESM1_ESM.pdf]

Surface-modified antiperovskite as electrocatalysts for water oxidation

Zhu *et al.*

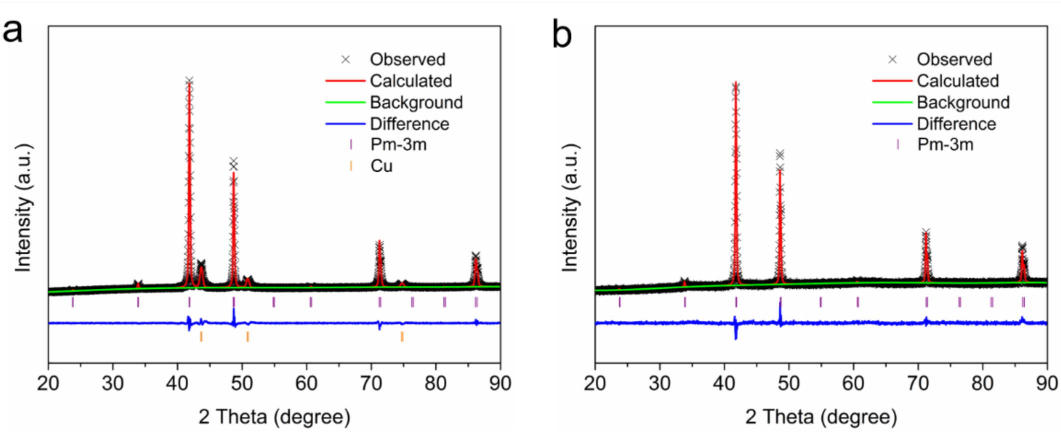

Supplementary Figure 1. Rietveld refinement XRD patterns. a $CuNNi_3$+Cu and **b** p-$Cu_{1-x}NNi_{3-y}$/FeNiCu.

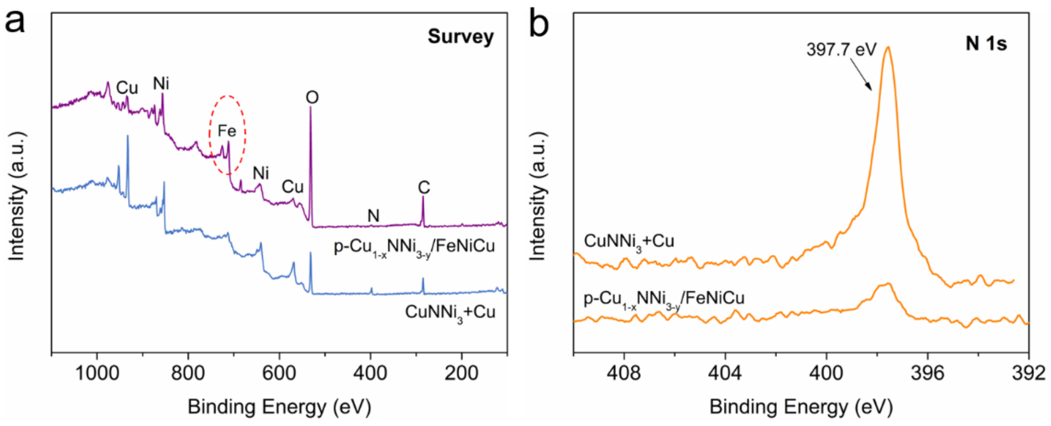

Supplementary Figure 2. XPS characterizations. a Survey and **b** N 1s XPS spectra of the $CuNNi_3$+Cu and the p-$Cu_{1-x}NNi_{3-y}$/FeNiCu.

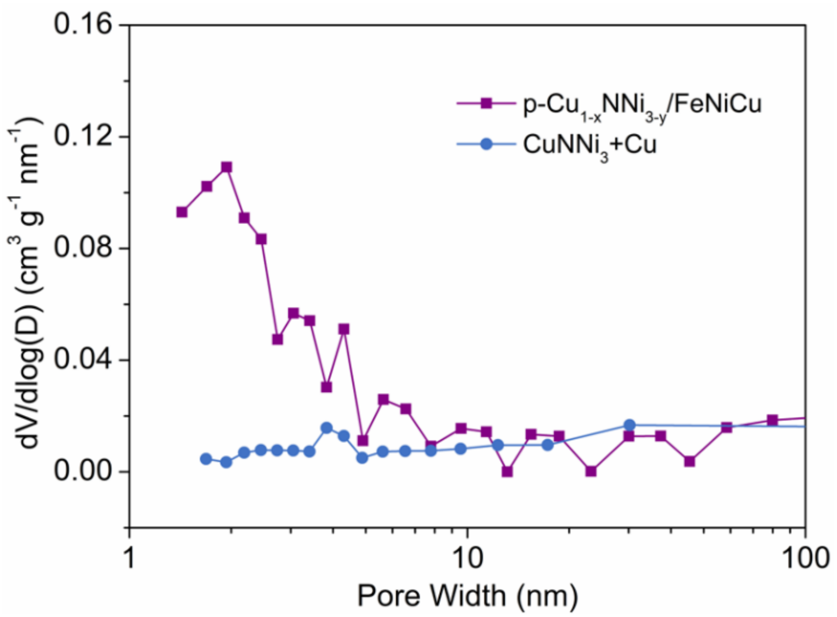

Supplementary Figure 3. Corresponding pore size distributions of $CuNNi_3$+Cu and p-$Cu_{1-x}NNi_{3-y}$/FeNiCu.

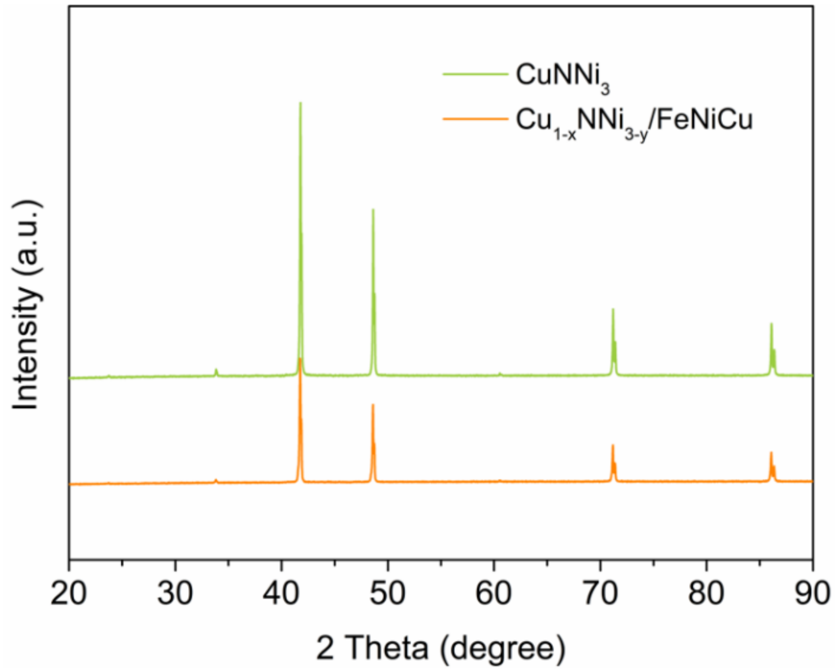

Supplementary Figure 4. XRD patterns of $CuNNi_3$ and $Cu_{1-x}NNi_{3-y}$/FeNiCu.

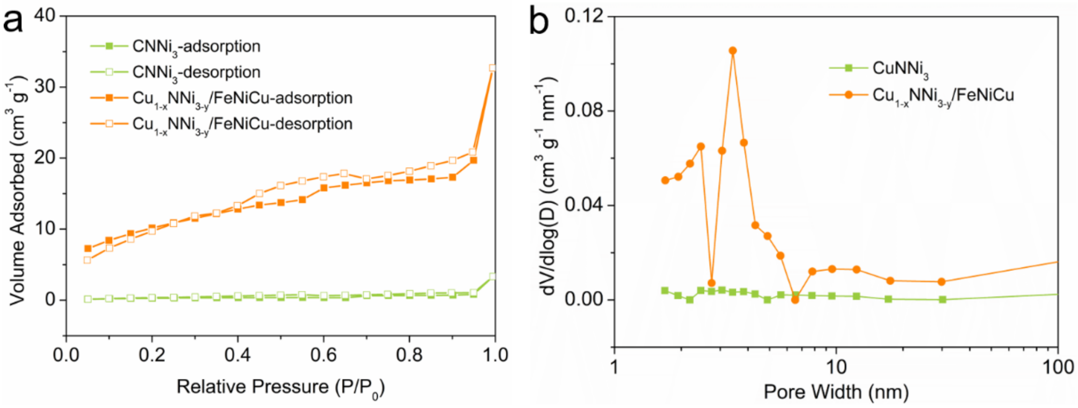

Supplementary Figure 5. Surface areas and pore size distributions. a N_2 adsorption-desorption isotherms and **b** the corresponding pore size distribution curves of $CuNNi_3$ and $Cu_{1-x}NNi_{3-y}$/FeNiCu.

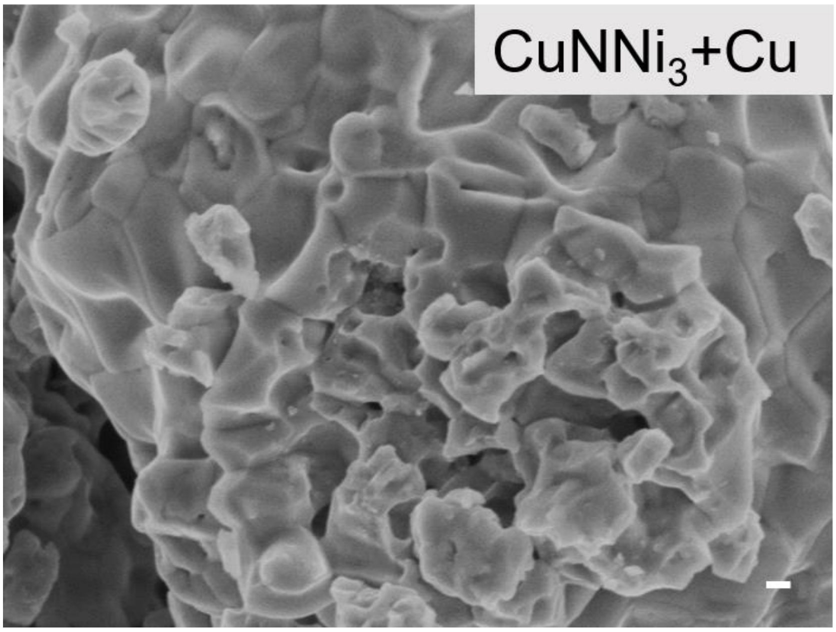

Supplementary Figure 6. SEM image of the $CuNNi_3$+Cu sample. Scale bar is 200 nm.

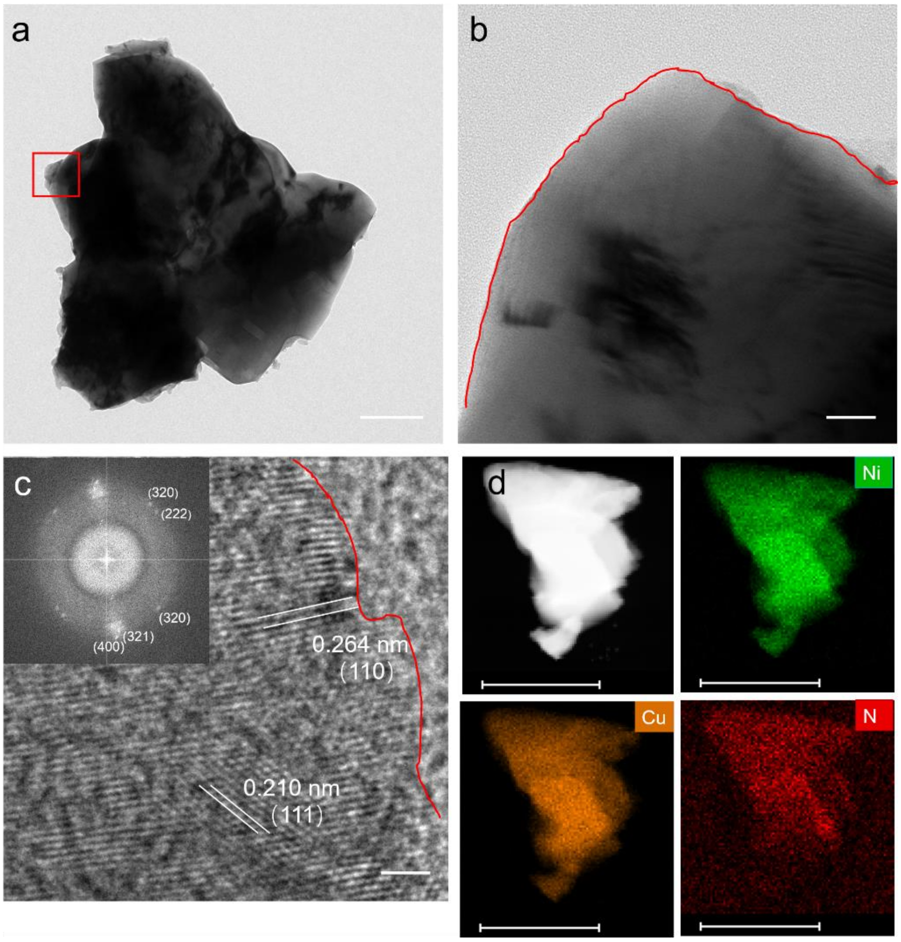

Supplementary Figure 7. Morphology and text characterization. a TEM and **b** magnified TEM images of the $CuNNi_3$+Cu. **c** HRTEM of the $CuNNi_3$+Cu. The inset is the corresponding FFT image. **d** STEM-EDS element mapping images of the $CuNNi_3$+Cu. Scale bar in **a** is 200 nm, in **b** is 20 nm, in **c** is 1 nm, in **d** is 500 nm.

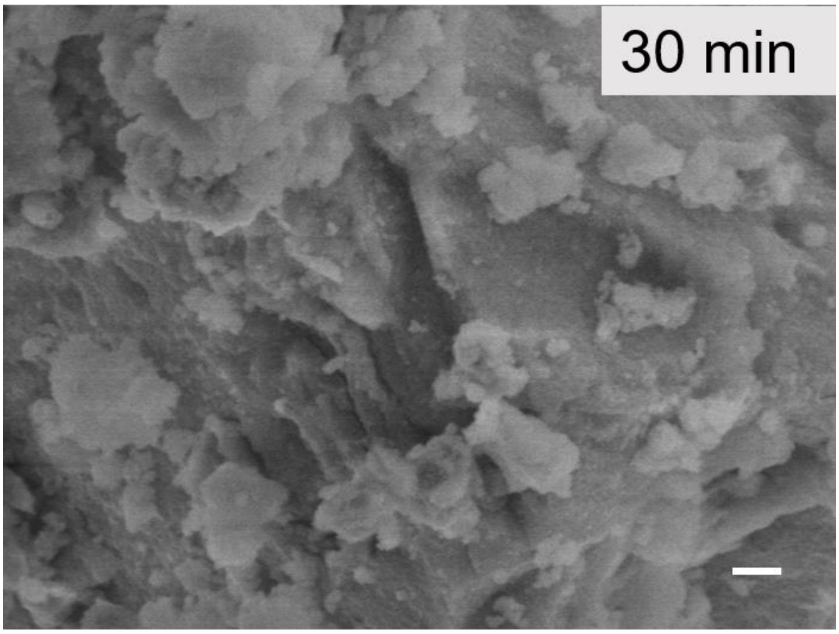

Supplementary Figure 8. SEM image of the p-$Cu_{1-x}NNi_{3-y}$/FeNiCu. Scale bar is 200 nm.

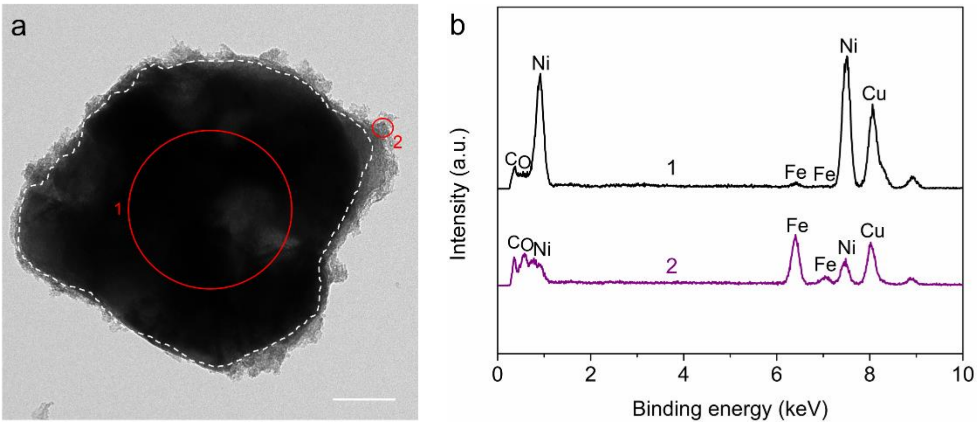

Supplementary Figure 9. TEM image and EDS spectra of p-$Cu_{1-x}NNi_{3-y}$/FeNiCu. a TEM image of p-$Cu_{1-x}NNi_{3-y}$/FeNiCu. **b** EDS spectra recorded from core (spot 1) and shell (spot 2). Scale bar in **a** is 100 nm.

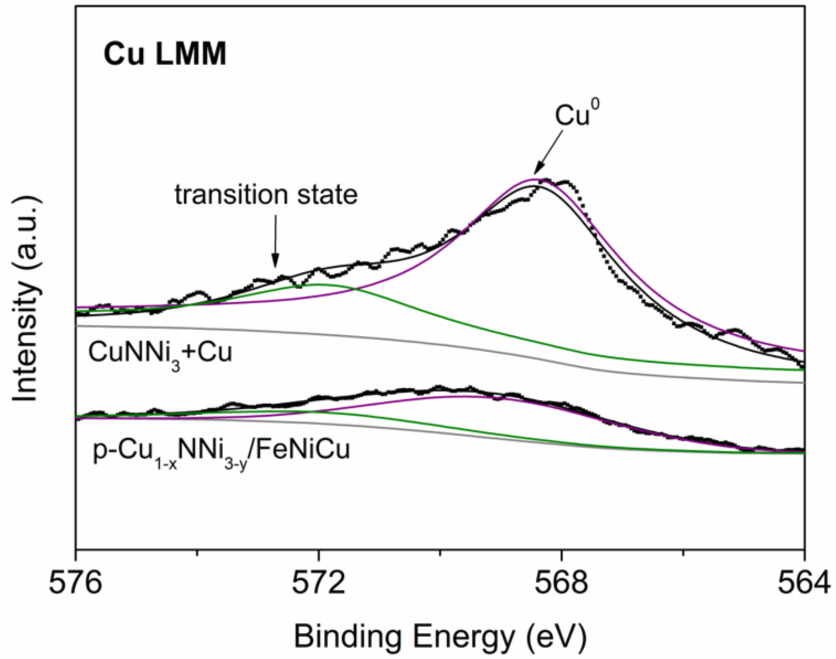

Supplementary Figure 10. XPS spectra of Cu LMM of the $CuNNi_3$+Cu and p-$Cu_{1-x}NNi_{3-y}$/FeNiCu.

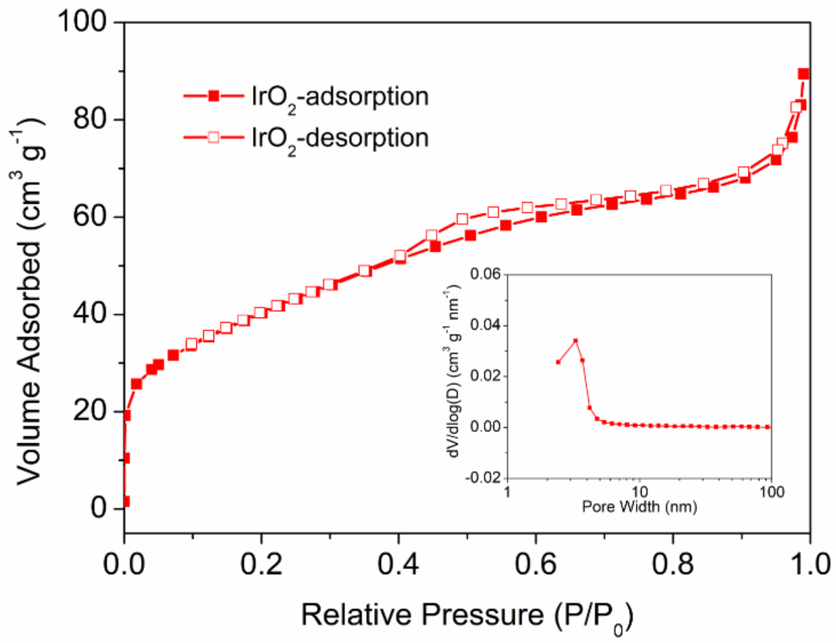

Supplementary Figure 11. Surface areas and pore size distributions. N_2 adsorption-desorption isotherms and the corresponding pore size distribution curves commercial IrO_2.

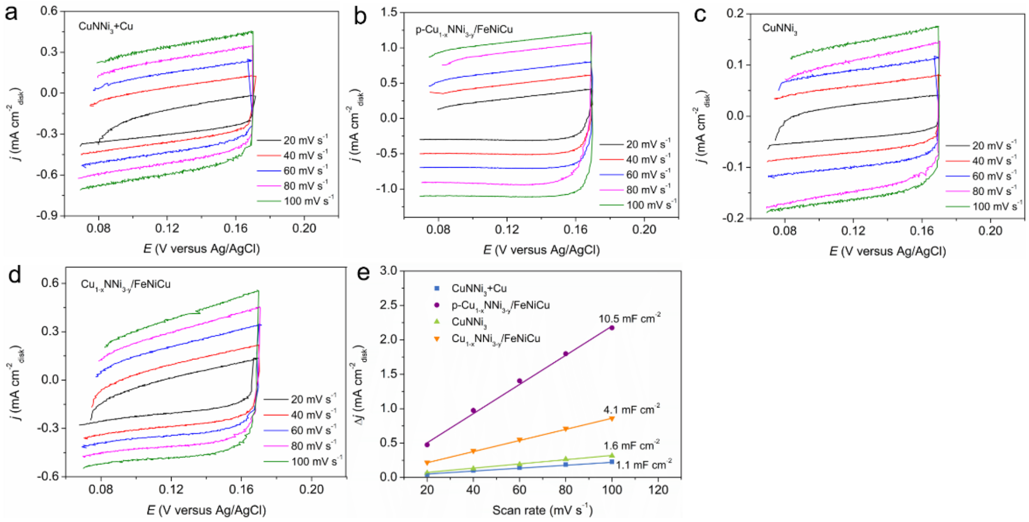

Supplementary Figure 12. ESCA measurement of samples. Electrochemical CV scans of **a** $CuNNi_3$+Cu, **b** p-$Cu_{1-x}NNi_{3-y}$/FeNiCu, **c** $CuNNi_3$ and **d** $Cu_{1-x}NNi_{3-y}$/FeNiCu. **e** the corresponding linear fitting of the capacitive currents versus CV scans.

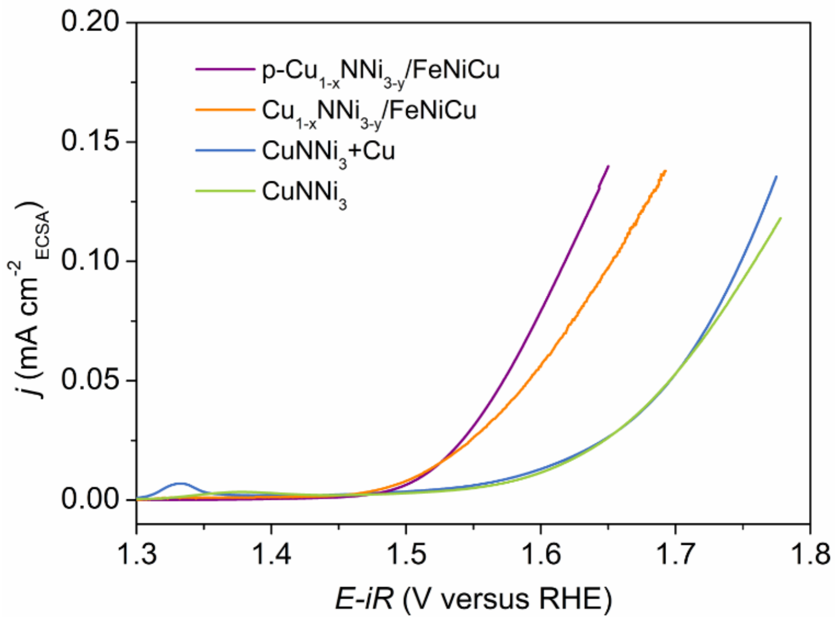

Supplementary Figure 13. Polarization curves normalized by ECSA for the $CuNNi_3$+Cu, p-$Cu_{1-x}NNi_{3-y}$/FeNiCu, $CuNNi_3$ and $Cu_{1-x}NNi_{3-y}$/FeNiCu catalysts in 1 M KOH.

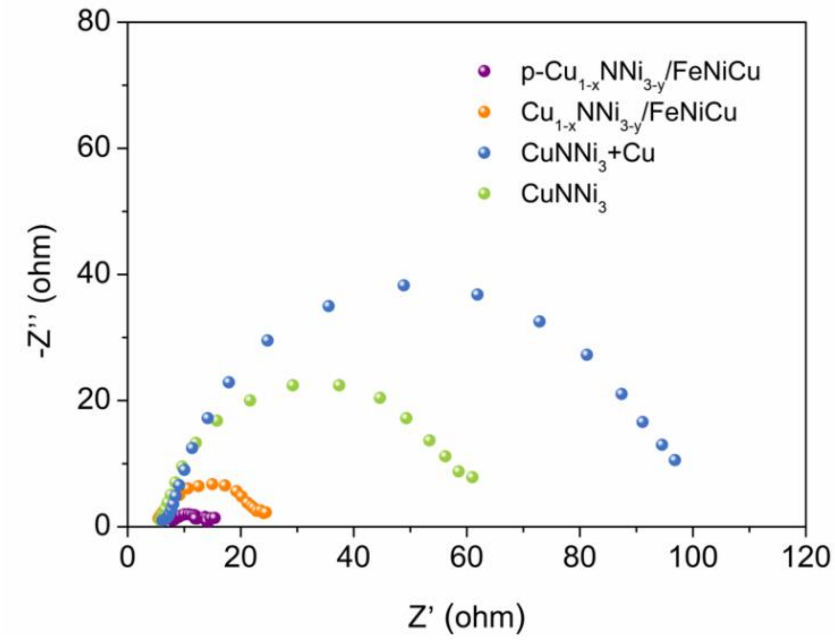

Supplementary Figure 14. EIS Nyquist plots of $CuNNi_3$+Cu, p-$Cu_{1-x}NNi_{3-y}$/FeNiCu, $CuNNi_3$ and $Cu_{1-x}NNi_{3-y}$/FeNiCu catalysts. The plots were collected 0.7 V versus Ag/AgCl under the influence of an AC voltage of 5 mV from 10^5 to 0.1 Hz.

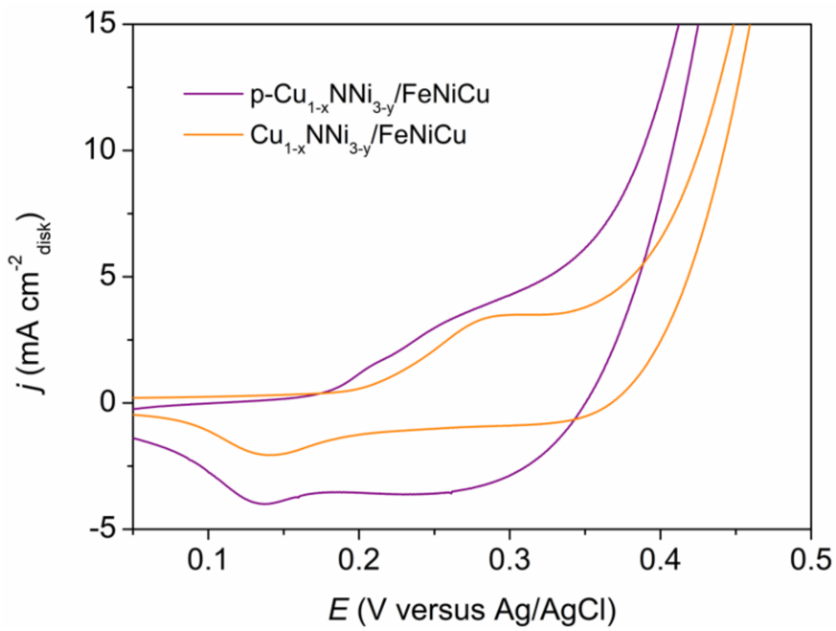

Supplementary Figure 15. Cyclic voltammograms of p-$Cu_{1-x}NNi_{3-y}$/FeNiCu and $Cu_{1-x}NNi_{3-y}$/FeNiCu electrodes in 6 M KOH.

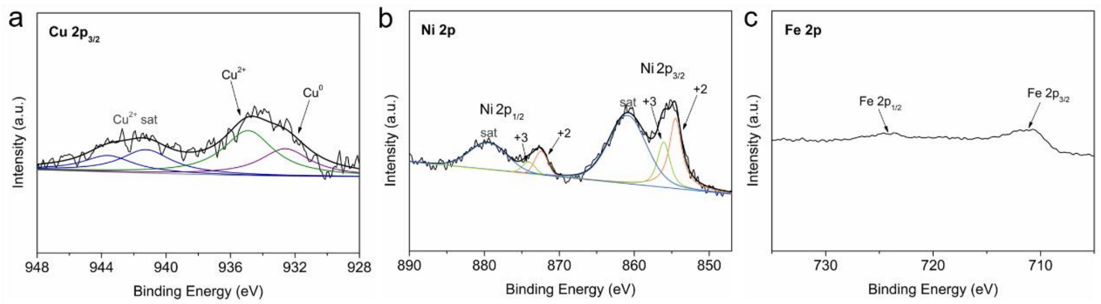

Supplementary Figure 16. XPS characterizations. **a** Cu $2p_{3/2}$, **b** Ni 2p and **c** Fe 2p XPS spectra of the p-$Cu_{1-x}NNi_{3-y}$/FeNiCu catalyst before and after OER at 1.5 V (versus RHE) for 1 h.

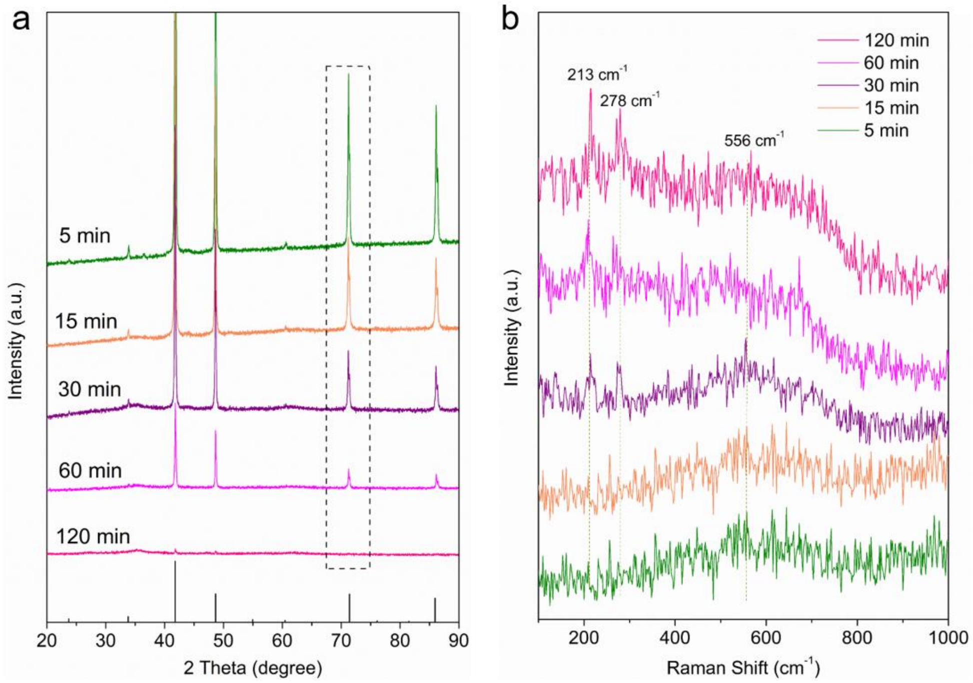

Supplementary Figure 17. XRD and Raman measurements. **a** XRD patterns and **b** Raman spectra of $Catalyst_{5min}$, $Catalyst_{15min}$, $Catalyst_{30min}$, $Catalyst_{60min}$ and $Catalyst_{120min}$.

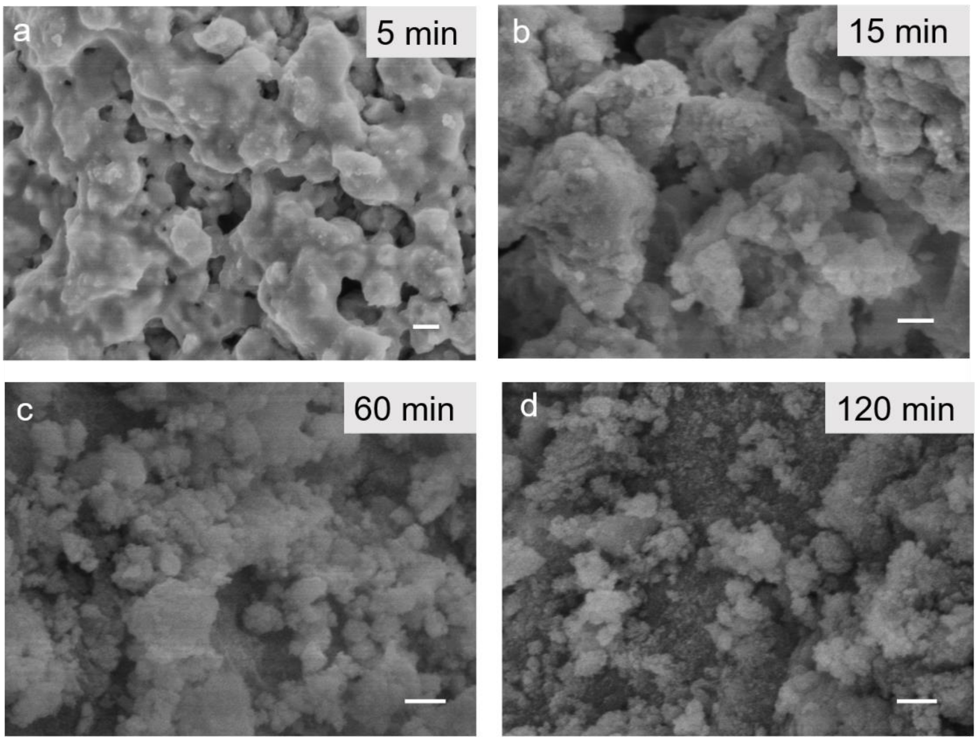

Supplementary Figure 18. SEM images. a Catalyst$_{5min}$, **b** Catalyst$_{15min}$, **c** Catalyst$_{60min}$ and **d** Catalyst$_{120min}$. Scale bar is 200 nm.

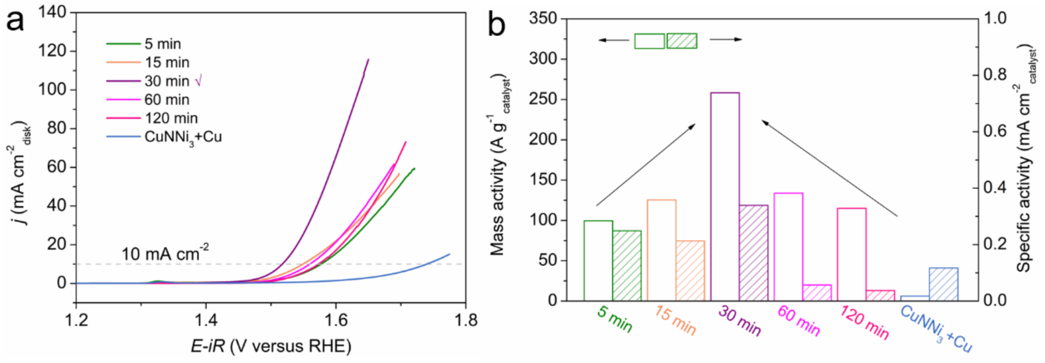

Supplementary Figure 19. OER activity curves. a Ohmic resistance-corrected OER activity curves of $CuNNi_3$+Cu, $Catalyst_{5min}$, $Catalyst_{15min}$, $Catalyst_{30min}$, $Catalyst_{60min}$ and $Catalyst_{120min}$ in 1 M KOH at 5 mV s^{-1} with a rotation rate of 1600 r.p.m. **b** Mass activities and BET surface area-normalized intrinsic activities of catalysts at η=400 mV derived from **a**.

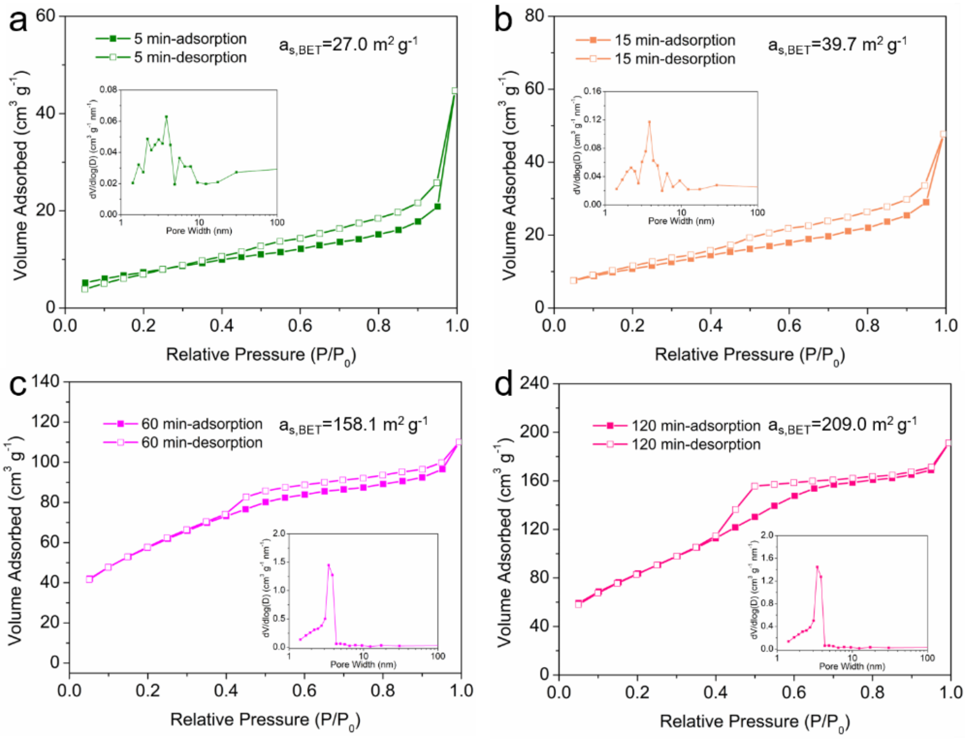

Supplementary Figure 20. Surface areas and pore size distributions. N_2 adsorption-desorption isotherms and the corresponding pore size distribution curves of **a** Catalyst$_{5min}$, **b** Catalyst$_{15min}$, **c** Catalyst$_{60min}$ and **d** Catalyst$_{120min}$.

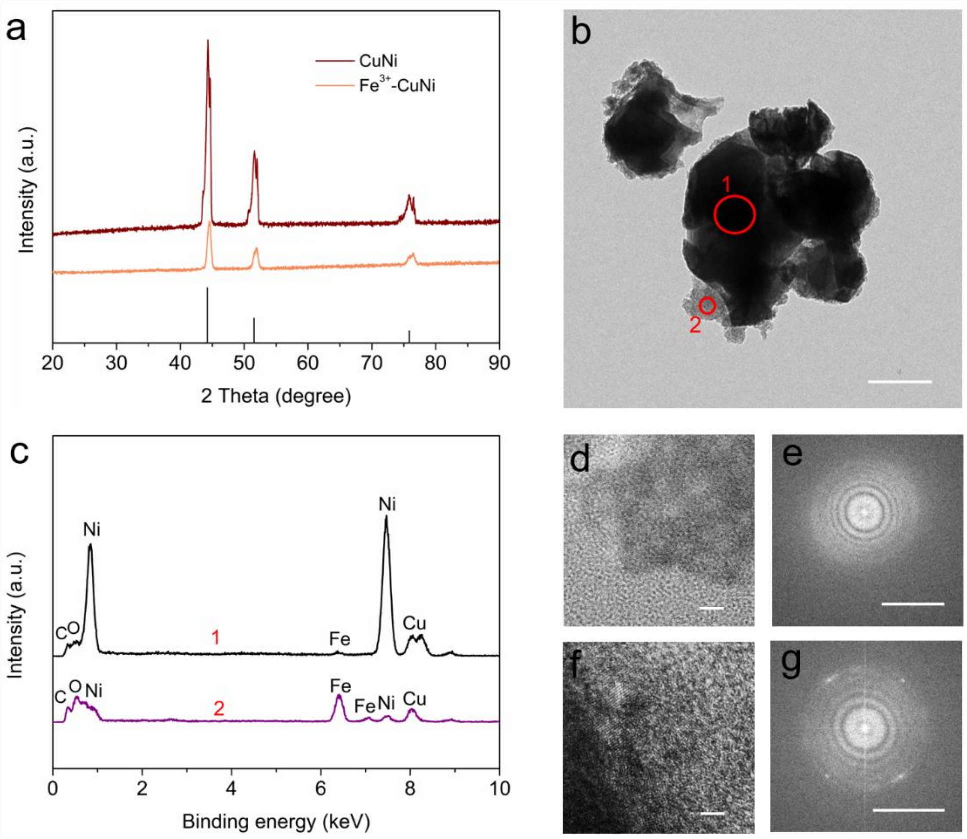

Supplementary Figure 21. Characterization of catalysts. a XRD patterns of CuNi and Fe^{3+}-CuNi. **b** TEM image of Fe^{3+}-CuNi. **c** EDS spectra recorded from core (spot 1) and shell (spot 2). **d** HRTEM and **e** the corresponding FFT images of the shell. **f** HRTEM and **g** the corresponding FFT images of the core. Scale bar in **b** is 200 nm, in **d** is 5 nm, in **e** is 5 nm^{-1}, in **f** is 5 nm, in **g** is 5 nm^{-1}.

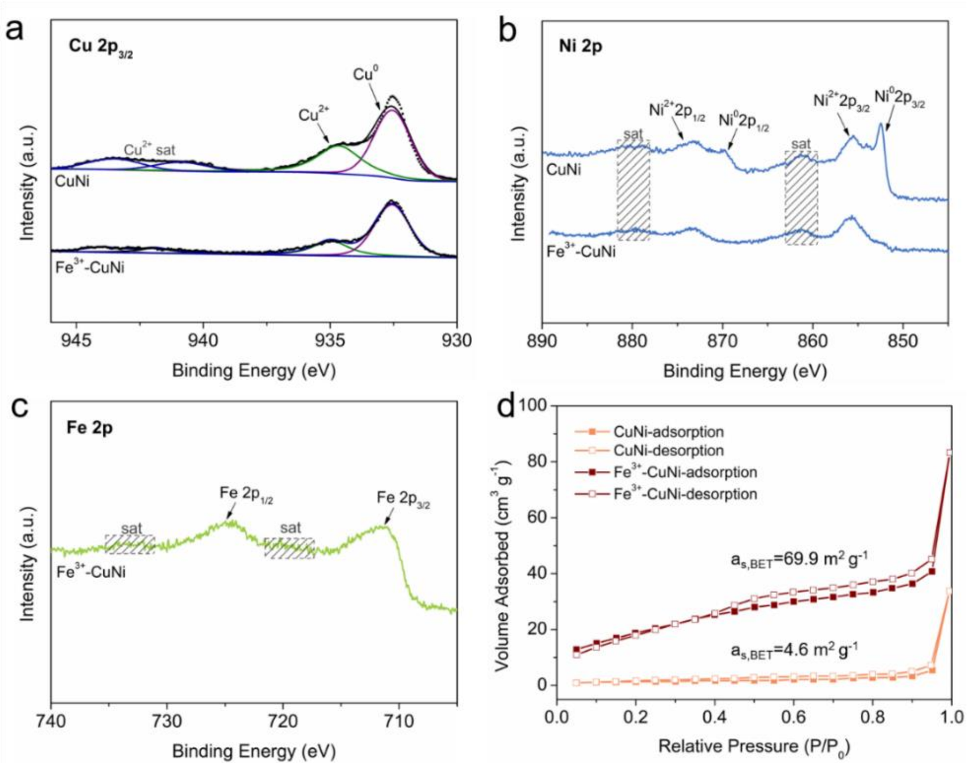

Supplementary Figure 22. Characterization of catalysts. XPS spectra of **a** Cu $2p_{3/2}$ and **b** Ni 2p of the CuNi and Fe^{3+}-CuNi. **c** Fe 2p XPS spectrum of the Fe^{3+}-CuNi. **d** N_2 adsorption-desorption isotherms of the CuNi and Fe^{3+}-CuNi.

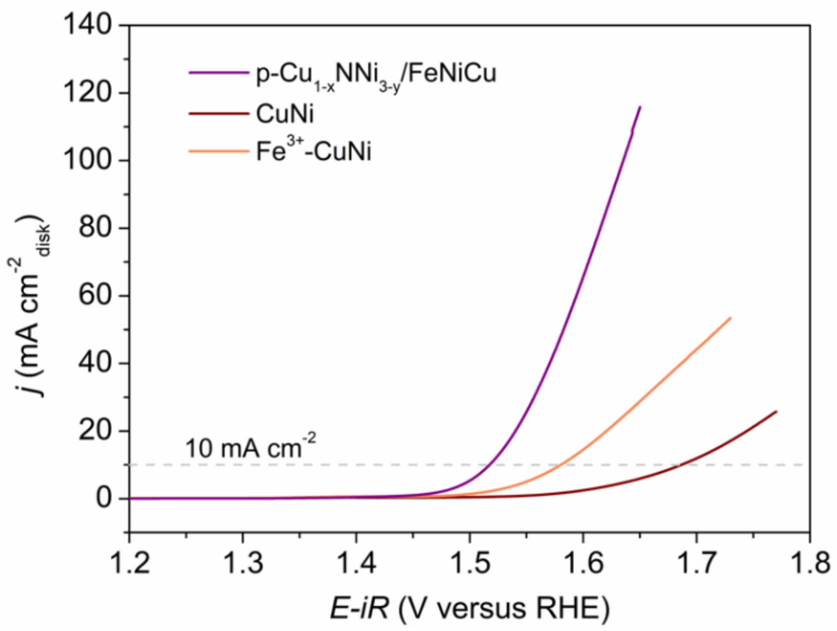

Supplementary Figure 23. OER activity curves of CuNi, Fe^{3+}-CuNi and p-$Cu_{1-x}NNi_{3-y}$/FeNiCu catalysts. The curves were recorded in 1 M KOH at 5 mV s^{-1} with a rotation rate of 1600 r.p.m.

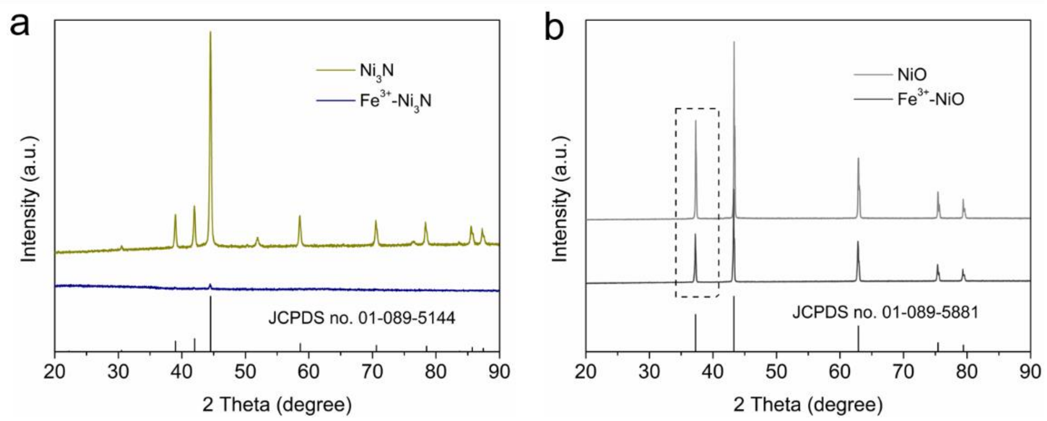

Supplementary Figure 24. XRD patterns. a Ni_3N and Fe^{3+}-Ni_3N and **b** NiO and Fe^{3+}-NiO.

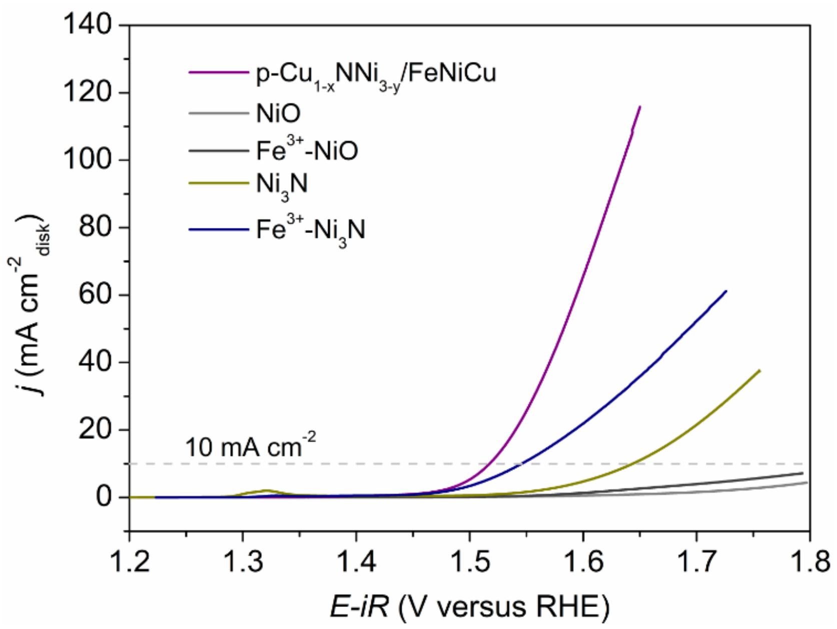

Supplementary Figure 25. OER activity curves of Ni_3N, Fe^{3+}-Ni_3N, NiO, Fe^{3+}-NiO and p-$Cu_{1-x}NNi_{3-y}$/FeNiCu catalysts. The curves were recorded in 1 M KOH at 5 mV s^{-1} with a rotation rate of 1600 r.p.m.

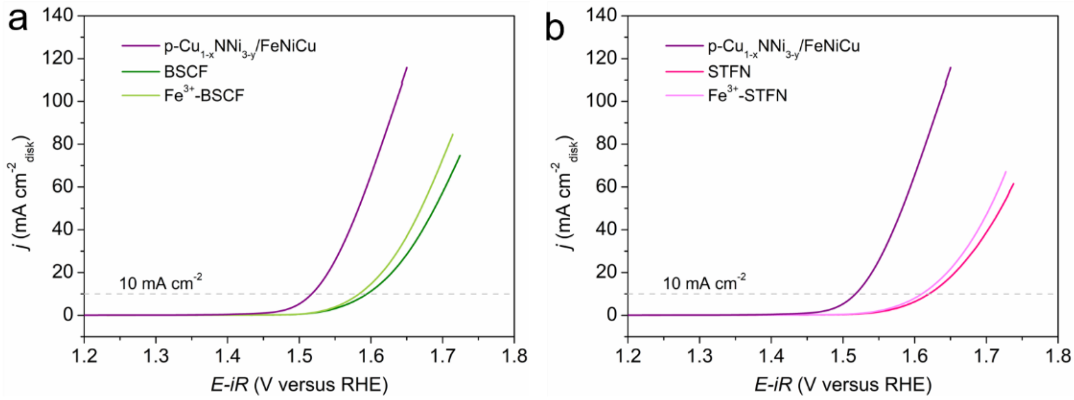

Supplementary Figure 26. OER activity curves. a BSCF and Fe^{3+}-BSCF, **b** STFN and Fe^{3+}-STFN. The curves were recorded in 1 M KOH at 5 mV s^{-1} with a rotation rate of 1600 r.p.m.

Supplementary Table 1. Rietveld refined lattice parameters and reliability factors for antiperovskites.

Sample	Lattice parameter (Å)	χ^2	R_p (%)	R_{wp} (%)
$CuNNi_3$+Cu	3.7410(5)	2.9269	2.12	2.95
p-$Cu_{1-x}NNi_{3-y}$/FeNiCu	3.7405(7)	1.573	2.33	2.99

Supplementary Table 2. Specific surface area (A_s) and total pore volumes of various materials.

Catalyst	A_s (m^2 g^{-1})	Total pore volume (cm^3 g^{-1})
$CuNNi_3$+Cu	5.2	0.004
**p-$Cu_{1-x}NNi_{3-y}$/FeNiCu**	**76.7**	**0.095**
$CuNNi_3$	3.5	0.006
$Cu_{1-x}NNi_{3-y}$/FeNiCu	36.9	0.058
$Catalyst_{5min}$	27.0	0.076
$Catalyst_{15min}$	39.7	0.078
$Catalyst_{60min}$	158.1	0.194
$Catalyst_{120min}$	209.0	0.167
IrO_2	141.8	0.112

Supplementary Table 3. Compositions of the samples from ICP-OES.

Samples	The concentrations of metal ions (mg L^{-1})		
	Cu	Ni	Fe
$CuNNi_3$+Cu	19.7	39.6	/
**p-$Cu_{1-x}NNi_{3-y}$/FeNiCu**	**8.92**	**23.1**	**11.1**
$CuNNi_3$	13.5	37.3	/
$Cu_{1-x}NNi_{3-y}$/FeNiCu	6.56	30.4	11.8

Supplementary Table 4. Comparison electrocatalytic OER activity in alkaline condition (1 M KOH) for p-$Cu_{1-x}NNi_{3-y}$/FeNiCu with some of the most active non-precious OER catalysts ever reported.

Catalyst	Substrate	η_{10} (mV)	Tafel slope (mV dec^{-1})	Reference
**p-$Cu_{1-x}NNi_{3-y}$/FeNiCu**	**GCE**	**280**	**52**	**This work**
NiFe-LDH	GCE	300	40	[1]
Ni_3S_2	Ni foam	157	159	[2]
FeNi-GO LDH	Ni foam	210	40	[3]
$CoSe_{0.85}$	CFC	324	85	[4]
Co_4N	GCE	330	58	[5]
Ni_3N nanosheets	GCE	350	85	[6]
CoMnP	GCE	330	61	[7]
Ni_2P	Ni foam	290	59	[8]
CNT/FeOOH	GCE	250	36	[9]
$CuCo_2S_4$	GCE	310	86	[10]
$Ni_xFe_{1-x}Se_2$-DO	Ni foam	190	28	[11]

Supplementary Note 1. ECSA calculation:

The electrochemical active surface area (ECSA) can be estimated using the capacitance (C). The specific capacitance for a flat surface is generally found to be in the range of 20~60 μF cm^{-2}. 40 μF cm^{-2} was used in the following calculations of the ECSA as literatures generally did [12,13].

The following formula was used to calculate ECSA:

$$\mathrm{ECSA} = \frac{\mathrm{C}}{40\ \mathrm{uF\ cm^{-2}\ per\ cm^{-2}}}$$

Supplementary References

1. Song, F. &Hu, X. L. Exfoliation of layered double hydroxides for enhanced oxygen evolution catalysis. *Nat. Commun.* **5,** 4477 (2014).
2. Zhou, W. J. *et al.* Ni_3S_2 nanorods/Ni foam composite electrode with low overpotential for electrocatalytic oxygen evolution. *Energy Environ. Sci.* **6,** 2921-2924 (2013).
3. Long, X. *et al.* A strongly coupled graphene and FeNi double yydroxide hybrid as an excellent electrocatalyst for the oxygen evolution reaction. *Angew. Chem. Int. Ed.* **126,** 7714-7718 (2014).
4. Xia, C., Jiang, Q., Zhao, C., Hedhili, M. N. & Alshareef, H. Selenide-base electrocatalysts and scaffolds for water oxidation applications. *Adv. Mater.* **28,** 77-85 (2016).
5. Xu, K. *et al.* Metallic nickel nitride nanosheets realizing enhanced electrochemical water oxidation. *J. Am. Chem. Soc.* **137,** 4119-4125 (2015).
6. Zhang, Y. Q. *et al.* Rapid synthesis of cobalt nitride nanowires: highly efficient and low-cost catalysts for oxygen evolution. *Angew. Chem. Int. Ed.* **55,** 8670-8674 (2016).
7. Li, D., Baydoun, H., Verani, C. N. & Brock, S. L. Efficient water oxidation using CoMnP nanoparticles. *J. Am. Chem. Soc.* **138,** 4006-4009 (2016).
8. Stern, L. A., Feng, L., Song, F. & Hu, X. L. Ni_2P as a Janus catalyst for water splitting: The oxygen evolution activity of Ni_2P nanoparticles. *Energy Environ. Sci.* **8,** 2347-2351 (2015).
9. Zhang, Y. Q. *et al.* Ultrathin CNTs@FeOOH nanoflake core/shell networks as efficient electrocatalysts for the oxygen evolution reaction. *Mater. Chem. Front.* **1,** 709-715 (2017).
10. Chauhan, M., Reddy, K. P., Gopinath, C. S. & Deka, S. Copper cobalt sulfide nanosheets realizing a promising electrocatalytic oxygen evolution reaction. *ACS Catal.* **7,** 5871-5879 (2017).
11. Xu, X., Song, F. & Hu, X. L. A nickel iron diselenide-derived efficient oxygen-evolution catalyst. *Nat. Commun.* **7,** 12324 (2016).
12. Chen, Y. Y. *et al.* Pomegranate-like N,P-doped Mo_2C@C nanospheres as highly active electrocatalysts for alkaline hydrogen evolution. *ACS Nano* **10,** 8851-8860 (2016).
13. Wang, X. D. *et al.* Novel porous molybdenum tungsten phosphide hybrid nanosheets on carbon cloth for efficient hydrogen evolution. *Energy Environ. Sci.* **9,** 1468-1475 (2016).
